# Effects of non-invasive neural stimulation modalities on upper limb function in subacute stroke: a systematic review and meta-analysis

**DOI:** 10.3389/fneur.2026.1805246

**Published:** 2026-05-29

**Authors:** Xiaoyan Liu, Kunning Wei, Fuyu Ye, Yanyan Wang, Chunli Mei

**Affiliations:** 1 Department of Nursing, Beihua University, Jilin, China; 2 Department of Nursing, Shandong University of Traditional Chinese Medicine, Jinan, China

**Keywords:** meta-analysis, non-invasive neural stimulation, rehabilitation, stroke, upper limb function

## Abstract

**Introduction:**

Non-invasive neural stimulation (NINS) has been increasingly adopted as an adjunctive strategy in stroke rehabilitation. However, its effectiveness in improving upper limb function during the subacute stage remains uncertain. This study aimed to evaluate the effects of different NINS modalities on upper limb function and activities of daily living in patients with subacute stroke.

**Methods:**

A systematic review and meta-analysis of randomized controlled trials (RCTs) was conducted. PubMed, Embase, Web of Science, Cochrane Library, and major Chinese databases (CNKI, Wanfang, VIP) were systematically searched from inception to October 2025. Eligible studies included adult patients with subacute stroke (7 d–6 m) receiving NINS. The primary outcomes were upper limb function and activities of daily living. Outcome data were extracted at the end of the intervention for quantitative synthesis. Standardized mean differences (SMDs) with 95% confidence intervals (CIs) were calculated using a random-effects model. Subgroup and meta-regression analyses prespecified in the study protocol were conducted based on recovery stage and stimulation modalities.

**Results:**

A total of 24 RCTs (*n* = 1,091 patients) were included. Compared with the control group, statistically substantial between-group differences were observed for FMA-UE [SMD = 0.60, 95% CI (0.30, 0.89)], Barthel Index [SMD = 0.77, 95% CI (0.42, 1.13)], ARAT [SMD = 0.66, 95% CI (0.40, 0.93)], and WMFT [SMD = 0.75, 95% CI (0.22, 1.27)] (all *p* < 0.05), whereas no substantial between-group differences were observed for BBT or MAS. Substantial heterogeneity was observed across outcomes (*I*^2^ range: 61.8–84.1%). Meta-regression did not identify substantial associations between effect size and recovery stage or stimulation modality (*p* > 0.05). Subgroup analyses showed substantial between-group differences in some modality-specific comparisons; however, no clear superiority was observed. In the transcranial direct current stimulation (tDCS) subgroup, a statistically substantial between-group difference in MAS scores was observed. Residual heterogeneity may reflect unmeasured confounders and limit the interpretability of pooled estimates.

**Conclusion:**

NINS may be associated with modest, context-dependent improvements in upper limb function and activities of daily living in patients with subacute stroke. However, given the substantial heterogeneity and methodological variability, current evidence remains limited and should be interpreted cautiously. Further large-scale, high-quality trials are required to clarify optimal stimulation modalities and clinical applicability.

**Systematic review registration:**

PROSPERO, CRD420251242784.

## 1 Introduction

Stroke continues to rank among the leading causes of long-term disability worldwide, placing a sustained and substantial burden on individuals and healthcare systems (1, 2). Recent evidence from the Global Burden of Disease study indicates that this burden is not static; rather, stroke-related disability has continued to increase (3, 4). Upper limb dysfunction represents one of the most prevalent and disabling sequelae following stroke, affecting an estimated 80% of patients during the early stages of recovery (3, 4). Recovery, when it occurs, is often partial. Functional restoration is shaped by a complex interplay of factors, including spasticity (5), lesion characteristics (6), and the timing and intensity of rehabilitation interventions (7).

Stroke recovery is typically described across four temporal phases: hyperacute (<24 h), acute (1–7 d), subacute (7 d–6 m), and chronic (>6 m) (8). Among these, the subacute stage has attracted considerable attention. Heightened neuroplasticity during this window is widely regarded as a key driver of functional restoration (9). Interventions delivered at this stage may facilitate cortical reorganization and support motor recovery (9, 10). Timing is therefore likely to play an important role in therapeutic responsiveness. The definition of the subacute stage (7 d–6 m) was adopted based on established clinical staging frameworks, with further stratification into early (7 d–3 m) and late (3–6 m) stages to ensure consistency in analysis (8).

Conventional rehabilitation for post-stroke upper limb dysfunction encompasses physical therapy, pharmacological management, constraint-induced movement therapy, and related interventions (7). Although these approaches are widely implemented in clinical practice, their effectiveness is not uniform. Patients with moderate to severe impairment often derive limited benefit (11), and certain interventions may be accompanied by adverse effects or challenges in adherence. This suggests that existing strategies, although indispensable, are not sufficient in isolation. Adjunctive therapies that are effective and well tolerated remain a clinical priority.

Non-invasive neural stimulation (NINS) has gained increasing attention as an adjunctive approach in stroke rehabilitation (12, 13). Its appeal lies in the capacity to modulate cortical excitability and reshape large-scale neural network dynamics—processes closely linked to neuroplasticity and motor recovery (14). These effects, however, are not mediated through a single pathway. Rather, they reflect a heterogeneous set of stimulation modalities. Central approaches, such as transcranial direct current stimulation (tDCS) and repetitive transcranial magnetic stimulation (rTMS) (15), act primarily at the cortical level. Peripheral approaches, including transcutaneous auricular vagus nerve stimulation (taVNS) (15), and transcutaneous electrical acupoint stimulation (TEAS) (16), primarily target peripheral pathways. These modalities differ substantially in their mechanisms of action, stimulation parameters, and neural targets, which may lead to variability in clinical effects. Accordingly, potential differences across stimulation modalities should be interpreted cautiously rather than as direct evidence of comparative effectiveness.

Previous systematic reviews have examined NINS; however, important gaps remain. Evidence specific to the subacute stage—when neuroplasticity is most active—is limited, and the clinical relevance of comparisons across stimulation modalities remains uncertain. Accordingly, this meta-analysis synthesized randomized controlled trials (RCTs) in patients with subacute stroke to evaluate the effects of NINS as an adjunct to conventional rehabilitation or sham stimulation on upper limb function and activities of daily living. Prespecified subgroup and meta-regression analyses were conducted to explore potential sources of heterogeneity, and avoid causal or comparative overinterpretation.

## 2 Methods

### 2.1 Study registration

This study was prospectively registered in the International Prospective Register of Systematic Reviews (PROSPERO; registration number: CRD420251242784). The design, conduct, and reporting of the study adhered to the Preferred Reporting Items for Systematic Reviews and Meta-Analyses guidelines (17).

### 2.2 Literature search strategy

A systematic search was conducted in major electronic databases, including PubMed, Embase, Web of Science, and the Cochrane Library, as well as Chinese databases such as China National Knowledge Infrastructure (CNKI), Wanfang, and VIP. All databases were searched from inception to October 2025.

The search strategy combined Medical Subject Headings and free-text terms. Core search terms included “stroke,” “upper limb,” and “non-invasive neural stimulation (NINS),” along with modality-related terms such as “electrical stimulation” and “magnetic stimulation.” In addition, specific stimulation modalities (e.g., tDCS, rTMS, and taVNS) and broader conceptual terms such as “stimulation” were incorporated. Both full terminology and commonly used abbreviations were included and combined using Boolean operators (AND/OR) to maximize sensitivity.

The search strategy was developed collaboratively by all authors prior to study selection. In addition, reference lists of included studies and relevant systematic reviews were manually screened to identify potentially eligible studies and minimize the risk of omission. The full PubMed search strategy is provided in Supplementary Table S1.

### 2.3 Eligibility criteria

Eligibility criteria were defined according to the PICOS framework: (1) Participants (P): Adults (≥18 years) diagnosed with stroke based on clinical criteria and confirmed by CT or MRI (18), in the subacute stage (7 d–6 m after stroke onset), with upper limb motor impairment; (2) Interventions (I): Any form of NINS; (3) Comparator (C): Conventional rehabilitation or sham stimulation; (4) Outcomes (O): At least one of the following: Fugl–Meyer Assessment for Upper Extremity (FMA-UE), Barthel Index, Action Research Arm Test (ARAT), Box and Block Test (BBT), Wolf Motor Function Test (WMFT), or Modified Ashworth Scale (MAS); (5) Study design (S): Randomized controlled trials (RCTs).

Exclusion criteria were as follows: (1) Full text unavailable; (2) Duplicate publications; (3) Insufficient or non-extractable data; (4) Non-original studies (e.g., reviews, meta-analyses, conference abstracts, or animal studies); (5) Absence of upper limb-related outcomes.

### 2.4 Study selection and data extraction

All retrieved records were imported into EndNote 21, and duplicates were removed prior to screening. Study selection and data extraction were conducted independently by two reviewers, with disagreements resolved by discussion or consultation with a third reviewer. Titles and abstracts were screened, followed by full-text assessment for eligibility.

Data were extracted using a standardized form, including study characteristics (first author, year, country, sample size), participant characteristics (age, sex, disease duration), intervention details (stimulation modalities and protocol), adverse events, and outcome measures. As all included studies were two-arm RCTs, no adjustment for multi-arm designs was required; if present, they would have been handled using established methods recommended by the Cochrane Handbook for Systematic Reviews of Interventions (19). When multiple post-intervention or follow-up time points were reported, the time point closest to the end of the intervention was selected to ensure comparability.

### 2.5 Risk of bias assessment

The risk of bias in included studies was assessed using the Cochrane Risk of Bias tool (20), covering the following domains: random sequence generation, allocation concealment, blinding of participants and personnel, blinding of outcome assessment, completeness of outcome data, selective reporting, and other sources of bias. The assessment was conducted independently by two reviewers, and discrepancies were resolved through discussion or consultation with a third reviewer when necessary. Each domain was classified as “low risk,” “high risk,” or “unclear risk” in accordance with established Cochrane guidelines. The results of the risk of bias assessment are presented in Supplementary Figures S1, S2.

### 2.6 Statistical analysis

Statistical analyses were performed using Stata/MP version 18.0. Continuous outcomes were pooled as standardized mean differences (SMDs) with 95% confidence intervals (CIs) based on post-intervention data. Outcome directions were harmonized to ensure consistency across measures.

Heterogeneity was assessed using the *I*^2^ statistic. A fixed-effects model was applied when *I*^2^ ≤ 50% and *p* > 0.10; otherwise, a random-effects model was used. Prespecified subgroup and meta-regression analyses were conducted based on recovery stage (7 d–3 m vs. 3–6 m) and stimulation modalities. Sensitivity analyses were performed using a leave-one-out approach.

Publication bias was evaluated using funnel plots and Egger’s test, with trim-and-fill applied where appropriate. A two-sided *p* < 0.05 was considered statistically substantial. Where available, minimal clinically important differences (MCIDs) were considered (21) (e.g., FMA-UE); otherwise, findings were interpreted cautiously. Given the clinical heterogeneity across stimulation modalities, pooled estimates were interpreted as overall adjunctive effects rather than comparative efficacy.

## 3 Results

### 3.1 Study selection

**Figure 1:**
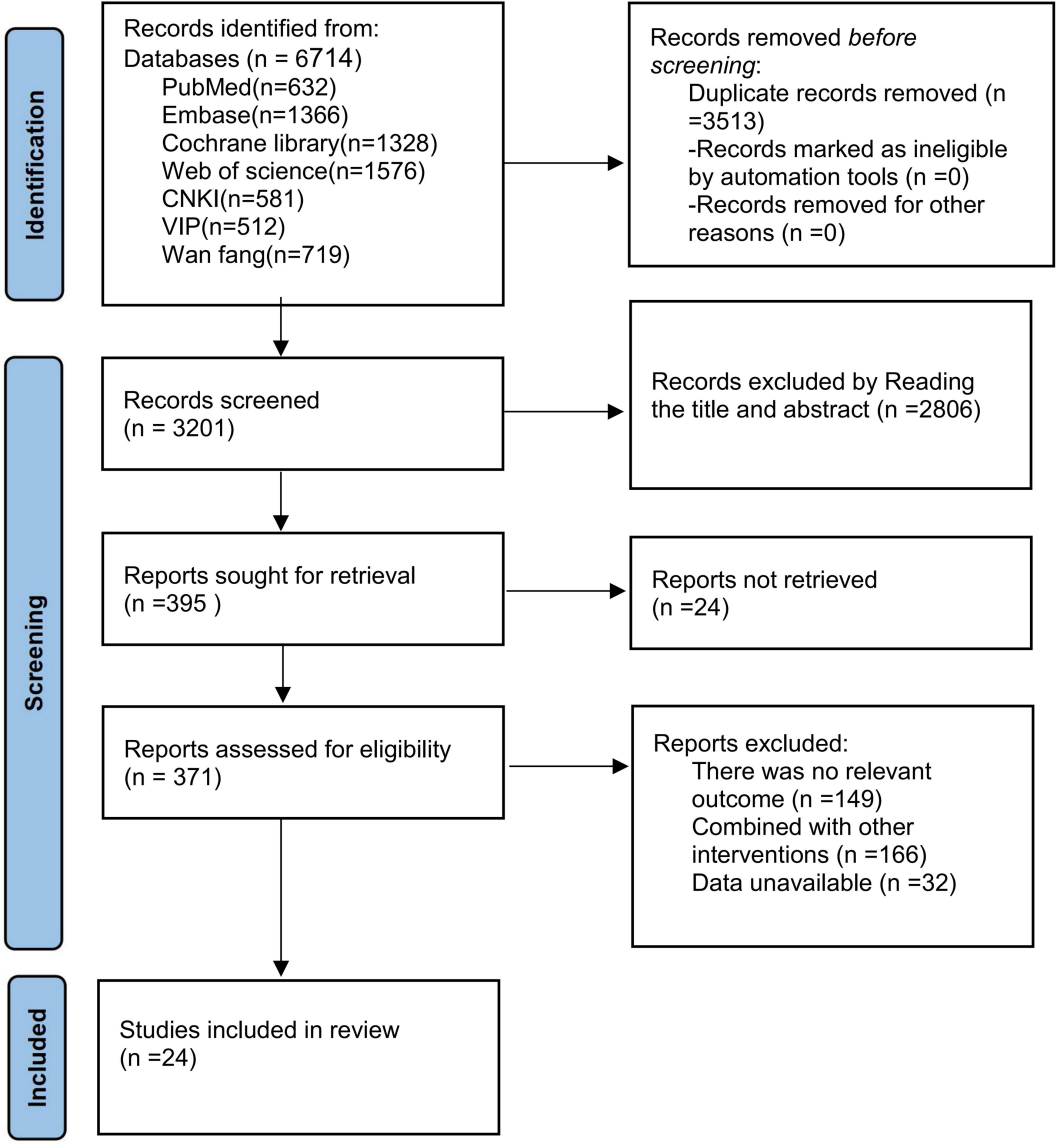
The flow chart of study selection. PRISMA 2020 flow diagram for new systematic reviews which included searches of databases and registers only. PRISMA (Preferred Reporting Items for Systematic Reviews and Meta-Analyses) flow diagram for studies included in and excluded from the meta-analysis. Consider, if feasible to do so, reporting the number of records identified from each database or register searched (rather than the total number across all databases/registers). If automation tools were used, indicate how many records were excluded by a human and how many were excluded by automation tools. From: Page et al. (17).

In total, 6,714 records were identified (4,902 in English and 1,812 in Chinese). After removal of duplicates, 3,201 records remained for screening. Of these, 2,806 were excluded after title and abstract screening, and a further 371 were excluded after full-text assessment. Ultimately, 24 RCTs involving NINS modalities were included in the final analysis (22–45). The study selection process is presented in Figure 1.

### 3.2 Study characteristics

A total of 24 RCTs involving 1,091 participants (553 in the intervention group and 538 in the control group) were included. Fifteen studies (22–36) were published in English, and nine in Chinese (37–45). The included trials evaluated a range of NINS modalities with varying stimulation protocols. Outcome measures primarily included FMA-UE (22, 23, 25–31, 33–45), Barthel Index (22, 24, 25, 27, 29, 30, 32, 35–38, 40–45), ARAT (22, 25, 28, 31, 35–39, 42–45), BBT (25–27, 30, 31), WMFT (23, 32, 41, 43, 44), and MAS (24, 38, 39). Regarding safety, among nine tDCS studies, three reported no adverse events (22, 41, 42); three reported mild adverse events, including tingling, itching, or erythema (25, 28, 40); and the remaining three did not report safety outcomes (38, 39, 45). Among six rTMS studies, one reported mild adverse events, including transient headache, dizziness, nausea, neck pain, sleep disturbances, anxiety, and attention deficits (32); one study did not report safety outcomes (27); and four studies reported no adverse events (26, 30, 31, 34). Other stimulation modalities, including intermittent theta-burst stimulation (iTBS), taVNS, transcutaneous electrical nerve stimulation, and repetitive peripheral magnetic stimulation (rPMS), were also well tolerated, with four studies not reporting safety data (24, 37, 43, 44); two studies reporting mild adverse events, such as headache, neck pain, muscle pain, nausea, burning sensation, or erythema (23, 35); and three studies reporting no adverse events (29, 33, 36). Overall, adverse events were mild, self-limiting, and infrequent, although reporting completeness varied across studies, highlighting the need for more systematic safety assessment in future research. Detailed study characteristics are presented in Table 1.

**Table 1:** The basic characteristics of the included studies.

Author	Year	Country	Sample size		Gender (M/F)	Mean age (years)		Time	Intervention		Adverse events	Outcomes
			EG	CG		EG	CG		EG	CG		
Gao et al. (22)	2025	China	20	19	27/12	57.00 ± 14.95	61.05 ± 11.58	3–6 months	Real tDCS + Conventional rehabilitation therapy	Sham tDCS + Conventional rehabilitation therapy	No adverse events	①②③
Pipatsrisawat et al. (23)	2022	Thailand	5	5	2/8	58.8 ± 5.9	59.2 ± 17.5	7 days–3 months	Real rTMS + cathodal tDCS + Conventional rehabilitation therapy	Sham rTMS + sham tDCS + Conventional rehabilitation therapy	Mild adverse events	①⑤
Moon et al. (24)	2021	South Korea	22	21	20/23	61.23 ± 7.24	61.62 ± 8.32	7 days–3 months	Real TENS + task-oriented training	Sham TENS + task-oriented training	Did not report	②⑥
Li et al. (25)	2024	China	26	26	42/10	58.65 ± 12.677	56.54 ± 10.428	3–6 months	Real tDCS + sensorimotor training	Sham tDCS + sensorimotor training	Mild adverse events	①②③④
Haghighi et al. (26)	2021	Iran	10	10	11/9	50.50 ± 9.47	53.90 ± 13.06	3–6 months	Real rTMS + Conventional rehabilitation therapy	Conventional rehabilitation therapy	No adverse events	①④
Shim et al. (27)	2023	South Korea	14	16	18/12	67.28 ± 10.80	63.56 ± 16.09	3–6 months	Real rTMS + motor learning	sham rTMS + motor learning	Did not report	①②④
Hsu et al. (28)	2023	China	13	14	15/12	59.1 ± 11.4	59.2 ± 11.8	7 days–3 months	Real tDCS + task-oriented training	Sham tDCS + task-oriented training	Mild adverse events	①③
Liu et al. (29)	2025	China	29	23	27/25	66	70.6	7 days–3 months	Real iTBS + Conventional rehabilitation therapy	Sham iTBS + Conventional rehabilitation therapy	No adverse events	①②
Chang et al. (30)	2010	South Korea	18	10	17/11	56.4	57.0	7 days–3 months	Real rTMS	Sham rTMS	No adverse events	①②④
Luk et al. (31)	2022	China	12	12	14/10	67.3	65.1	3–6 months	Real rTMS + motor task practice	Sham rTMS + motor task practice	No adverse events	①③④
Zheng et al. (32)	2015	China	58	54	68/44	65.4 ± 13.5	66.2 ± 13.1	7 days–3 months	Real rTMS + VR + conventional rehabilitation therapy	Sham rTMS + VR + Conventional rehabilitation therapy	Mild adverse events	②⑤
Ke et al. (33)	2022	China	13	13	14/12	58	56	7 days–3 months	Real rPMS + conventional rehabilitation therapy	Sham rPMS + Conventional rehabilitation therapy	No adverse events	①
Ibrahim et al. (34)	2020	Egypt	20	20	24/16	58.70 ± 5.58	60.20 ± 5.33	7 days–3 months	Real rTMS	Sham rTMS	No adverse events	①
Vink et al. (35)	2023	Netherlands	28	31	40/19	56.8 ± 12	63.4 ± 12	7 days–3 months	Real cTBS + conventional rehabilitation therapy	Sham cTBS + conventional rehabilitation therapy	Mild adverse events	①②③
Wang et al. (36)	2024	China	20	20	33/7	55 ± 11	57 ± 11	3–6 months	Real taVNS + task-oriented training	Sham taVNS + task-oriented training	No adverse events	①②③
Zou et al. (37)	2025	China	30	30	33/27	53.97 ± 10.18	53.93 ± 9.91	3–6 months	Real taVNS + dual-task training	Sham-taVNS + dual-task training	Did not report	①②③
Yin et al. (38)	2015	China	40	40	57/23	55.70 ± 12.32	57.68 ± 13.54	7 days–3 months	Real tDCS + conventional rehabilitation therapy	Sham tDCS + conventional rehabilitation therapy	Did not report	①②③⑥
Zeng et al. (39)	2023	China	25	25	30/20	59.88 ± 13.54	56.12 ± 12.16	7 days–3 months	Real tDCS + robot + conventional rehabilitation therapy	Sham tDCS + robot + conventional rehabilitation therapy	Didn’t report	①③⑥
Liu et al. (40)	2023	China	20	20	25/15	58.32 ± 10.28	61.09 ± 6.96	7 days–3 months	Real tDCS + conventional rehabilitation therapy	Sham tDCS + conventional rehabilitation therapy	Mild adverse events	①②
Wang et al. (41)	2025	China	28	28	29/27	53.11 ± 9.98	52.93 ± 10.76	3–6 months	Real tDCS + upper limb robot training + conventional rehabilitation therapy	Sham tDCS + upper limb robot training + conventional rehabilitation therapy	No adverse events	①②⑤
Pang et al. (42)	2023	China	33	32	30/35	63.93 ± 4.98	64.52 ± 5.21	7 days–3 months	Real tDCS + mirror therapy + conventional rehabilitation therapy	Sham tDCS + mirror therapy + conventional rehabilitation therapy	No adverse events	①②③
Xia et al. (43)	2021	China	24	24	35/13	56.20 ± 9.91	56.50 ± 7.90	7 days–3 months	Real TEAS + mirror therapy + conventional rehabilitation therapy	Sham TEAS + mirror therapy + conventional rehabilitation therapy	Did not report	①②③⑤
Chen et al. (44)	2025	China	25	25	30/20	68.00 ± 11.90	66.48 ± 11.72	3–6 months	Real iTBS + conventional rehabilitation training	Sham iTBS + conventional rehabilitation training	Did not report	①②③⑤
Sui et al. (45)	2022	China	20	20	31/9	61 ± 11	62 ± 12	3–6 months	Real tDCS + VR + conventional rehabilitation therapy	Sham tDCS + VR + conventional rehabilitation therapy	Did not report	①②③

EG, experimental group; CG, control group.

① FMA-UE, Fugl–Meyer Assessment of the Upper Extremity; ② Barthel Index; ③ ARAT, Action Research Arm Test; ④ BBT, Box and Block test; ⑤ WMFT, Wolf Motor Function Test; ⑥ MAS, Motor Assessment Scale.

tDCS, transcranial direct current stimulation; rTMS, repetitive transcranial magnetic stimulation; TEAS, transcutaneous electrical acupoint stimulation; iTBS, intermittent theta burst stimulation; rPMS, repetitive peripheral magnetic stimulation; cTBS, continuous theta burst stimulation; taVNS, transcutaneous auricular vagus nerve stimulation; TEAS, transcutaneous electrical acupoint stimulation.

### 3.3 Risk of bias

Most studies reported adequate random sequence generation (21/24) (22–25, 27–32, 35–45), whereas allocation concealment was described in only six studies (22–24, 31, 35, 44). Blinding of participants and personnel was reported in most trials (23/24) (22–29, 31–45), and blinding of outcome assessors was reported in 21 of 24 studies (22–26, 28, 30–44). All studies provided complete outcome data (22–45), and no clear evidence of selective reporting was identified. However, insufficient reporting in several trials resulted in multiple domains being rated as “unclear risk.” Detailed risk of bias assessments are presented in Supplementary Figures S1, S2.

### 3.4 Meta-analysis

Outcomes were categorized into impairment-level measures (e.g., FMA-UE, MAS) and functional outcomes (e.g., Barthel Index, ARAT, WMFT, BBT) to reflect different domains of recovery.

#### 3.4.1 FMA-UE

**Figure 2:**
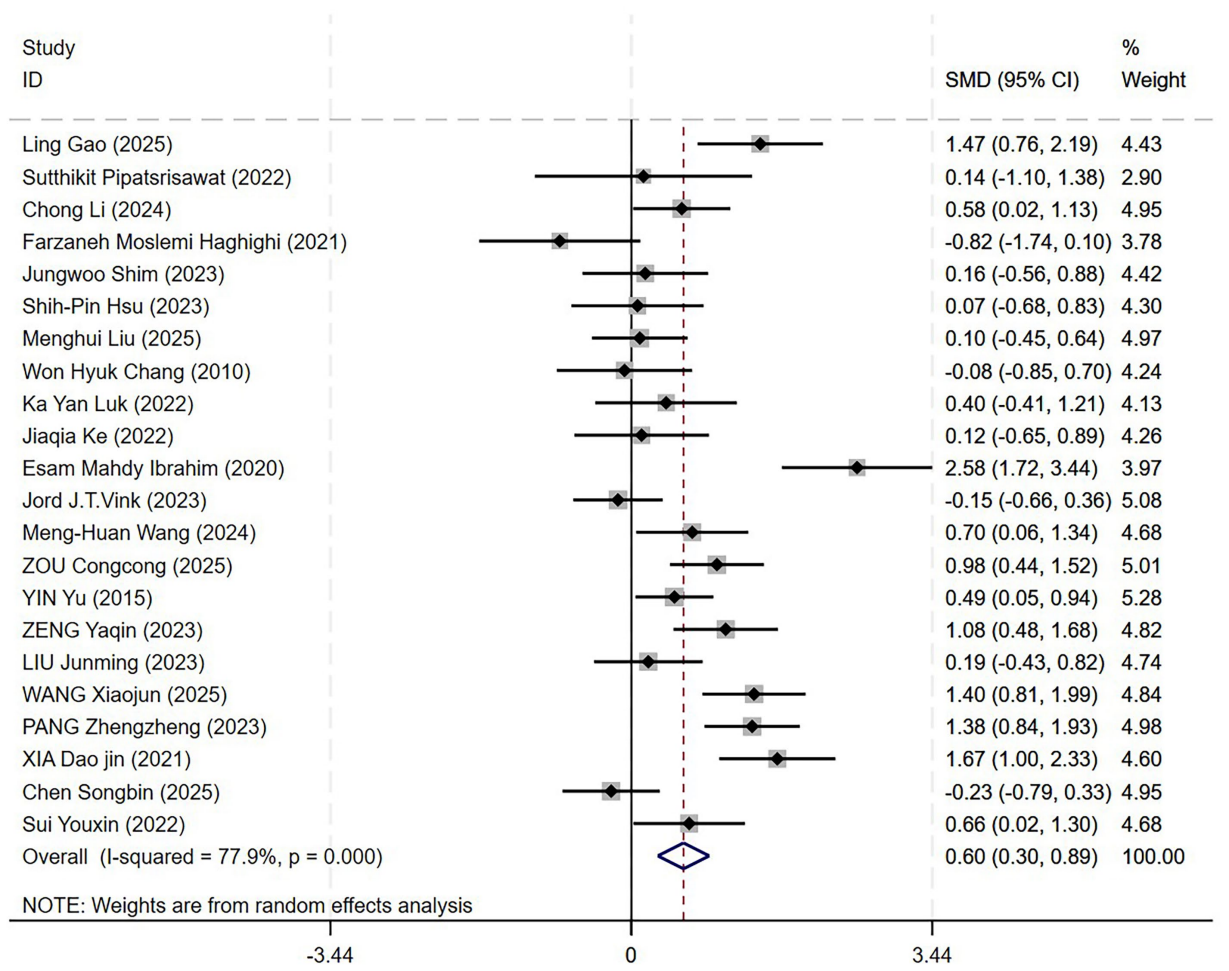
Forest plot of FMA-UE in subacute stroke. Squares indicate study weight; the diamond represents the overall effect; positive values favor the experimental group.

Twenty-two studies (22, 23, 25–31, 33–45) (*n* = 936) were included. Compared with the control group, the intervention group receiving NINS showed a statistically substantial difference in upper limb function based on post-intervention data [SMD = 0.60, 95% CI (0.30, 0.89), *p* < 0.05], although substantial heterogeneity was observed (*I*^2^ = 77.9%) (Figure 2).

#### 3.4.2 Barthel Index

**Figure 3:**
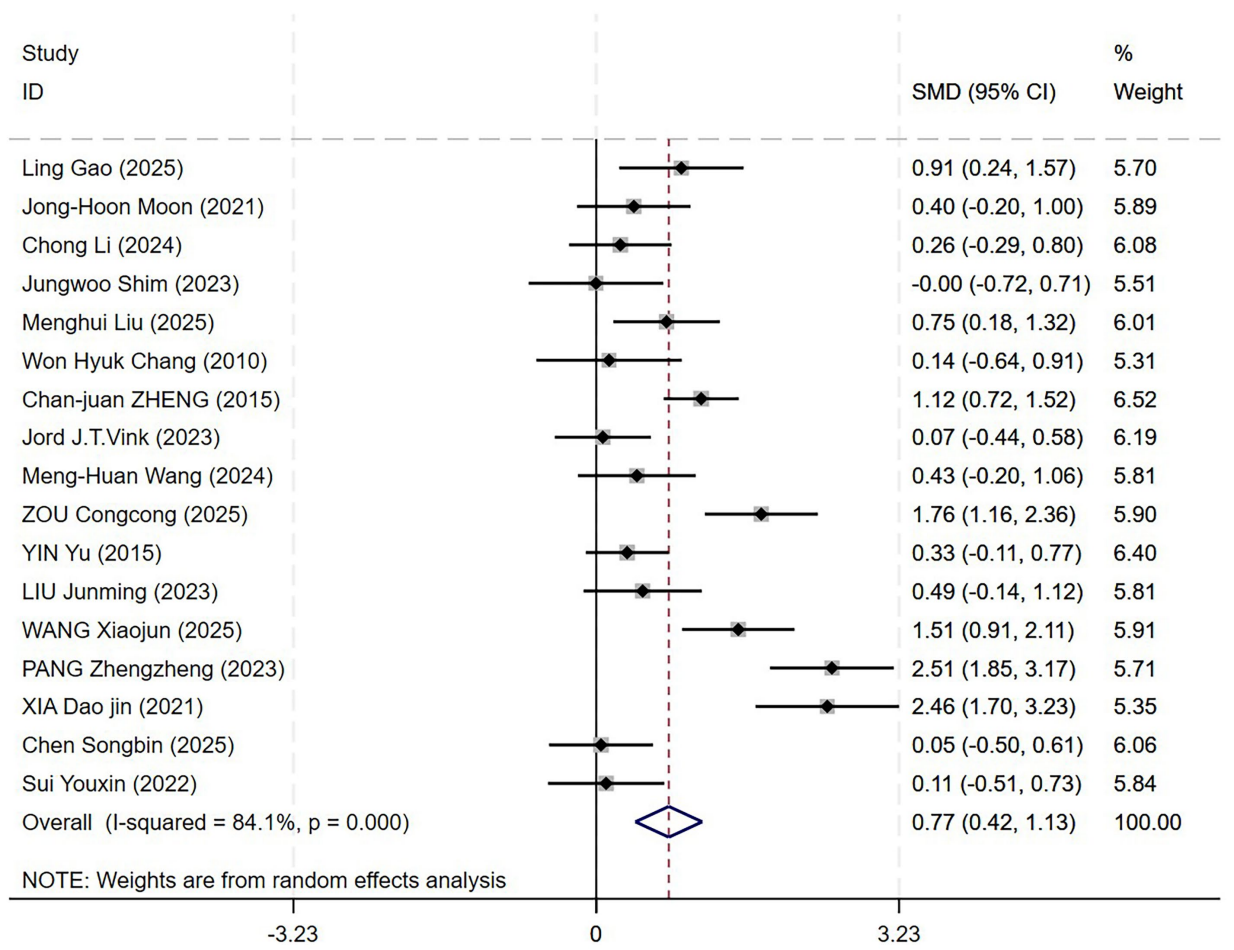
Forest plot of Barthel Index in subacute stroke. Squares indicate study weight; the diamond represents the overall effect; positive values favor the experimental group.

Seventeen studies (22, 24, 25, 27, 29, 30, 32, 35–38, 40–45) (*n* = 894) were included. NINS was associated with a statistically substantial between-group difference in activities of daily living [SMD = 0.77, 95% CI (0.42, 1.13), *p* < 0.05], with substantial heterogeneity (*I*^2^ = 84.1%) (Figure 3).

#### 3.4.3 ARAT

**Figure 4:**
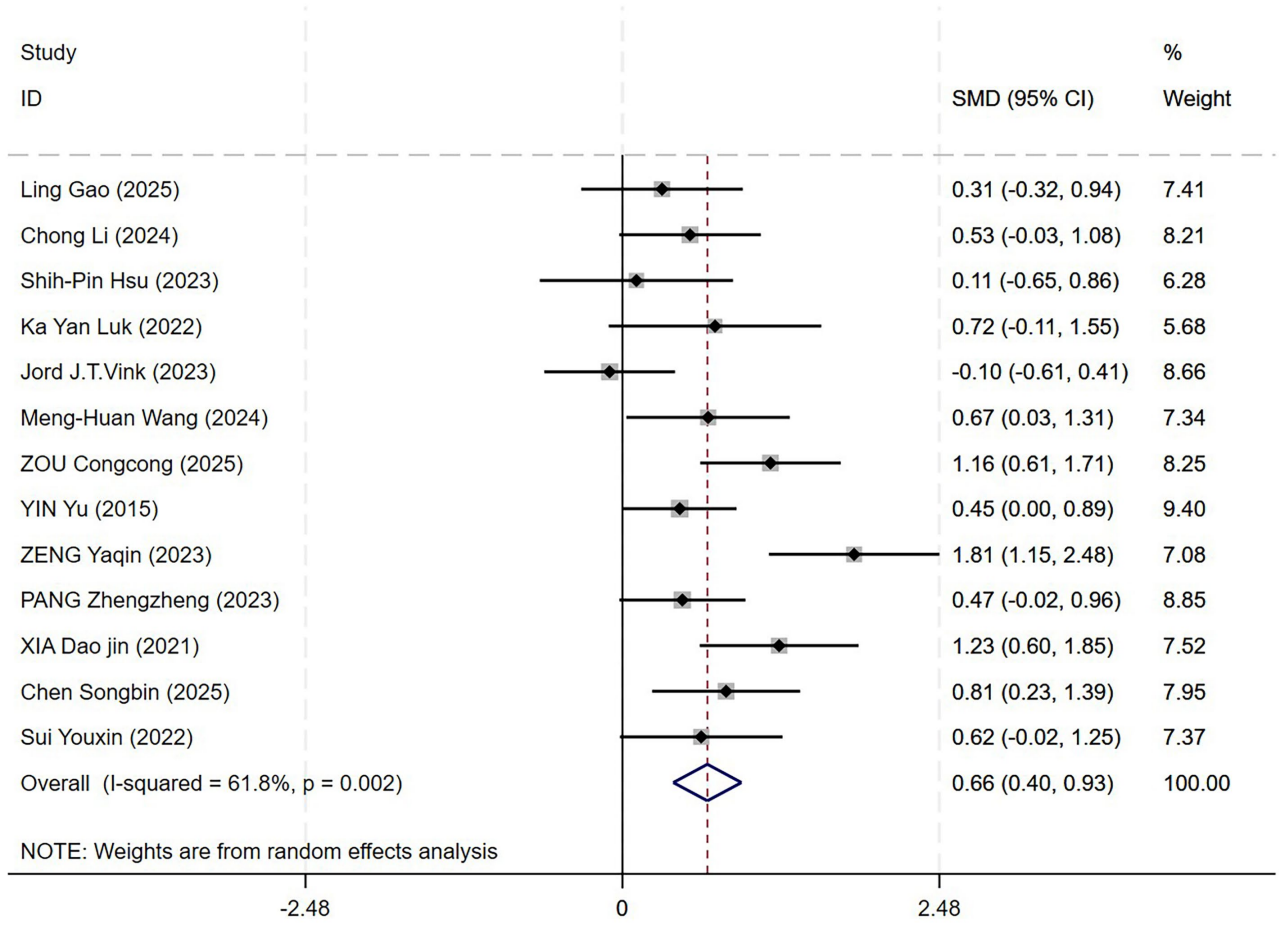
Forest plot of ARAT in subacute stroke. Squares indicate study weight; the diamond represents the overall effect; positive values favor the experimental group.

Thirteen studies (22, 25, 28, 31, 35–39, 42–45) (*n* = 634) were included. The intervention group showed a statistically substantial between-group difference in ARAT scores [SMD = 0.66, 95% CI (0.40, 0.93), *p* < 0.05], with moderate to substantial heterogeneity (*I*^2^ = 61.8%) (Figure 4).

#### 3.4.4 BBT

**Figure 5:**
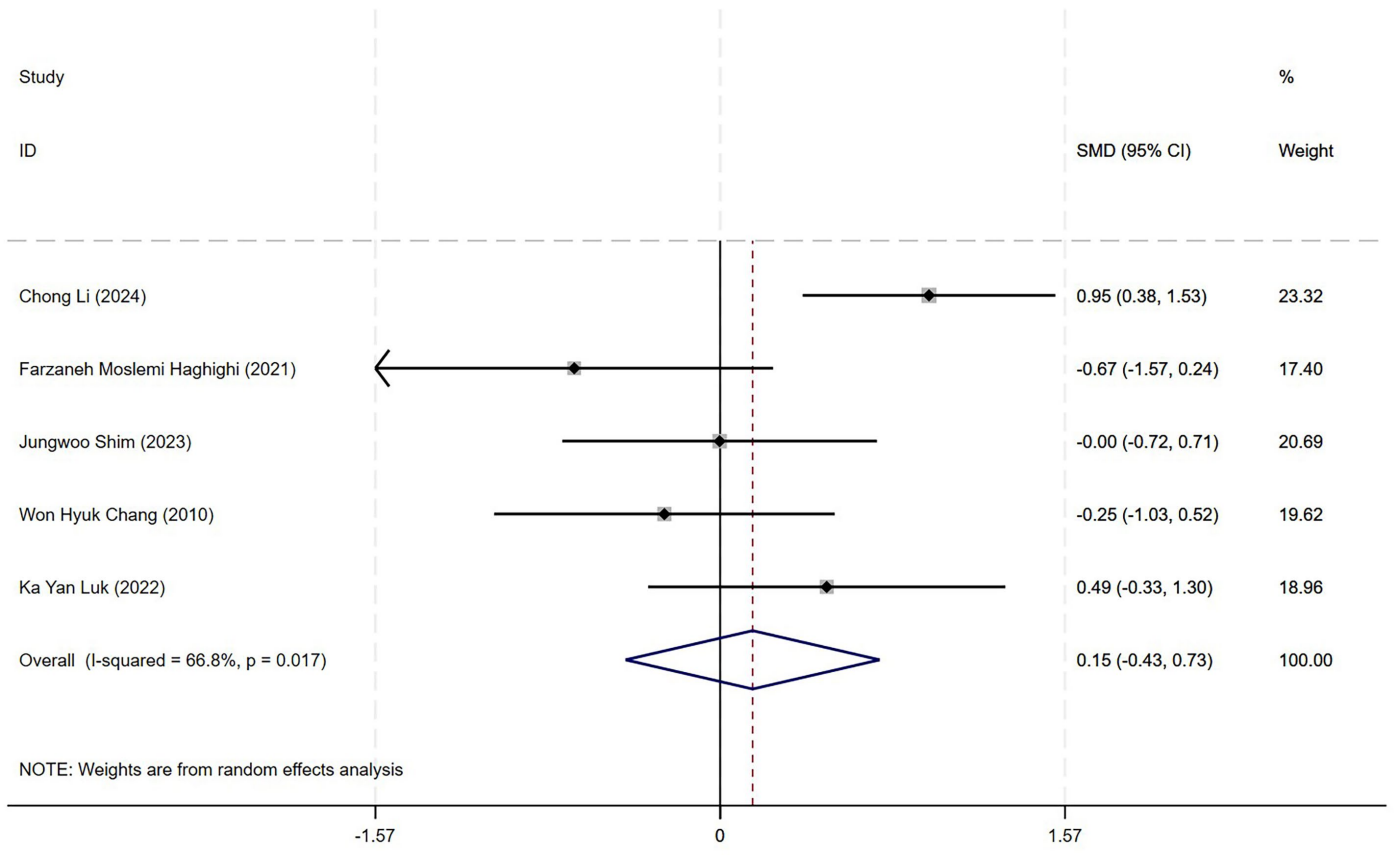
Forest plot of BBT in subacute stroke. Squares indicate study weight; the diamond represents the overall effect; positive values favor the experimental group.

Five studies (25–27, 30, 31) (*n* = 154) were included. No statistically substantial between-group difference was observed in BBT scores based on post-intervention data [SMD = 0.15, 95% CI (−0.43, 0.73), *p* = 0.617], with substantial heterogeneity (*I*^2^ = 66.8%) (Figure 5).

#### 3.4.5 WMFT

**Figure 6:**
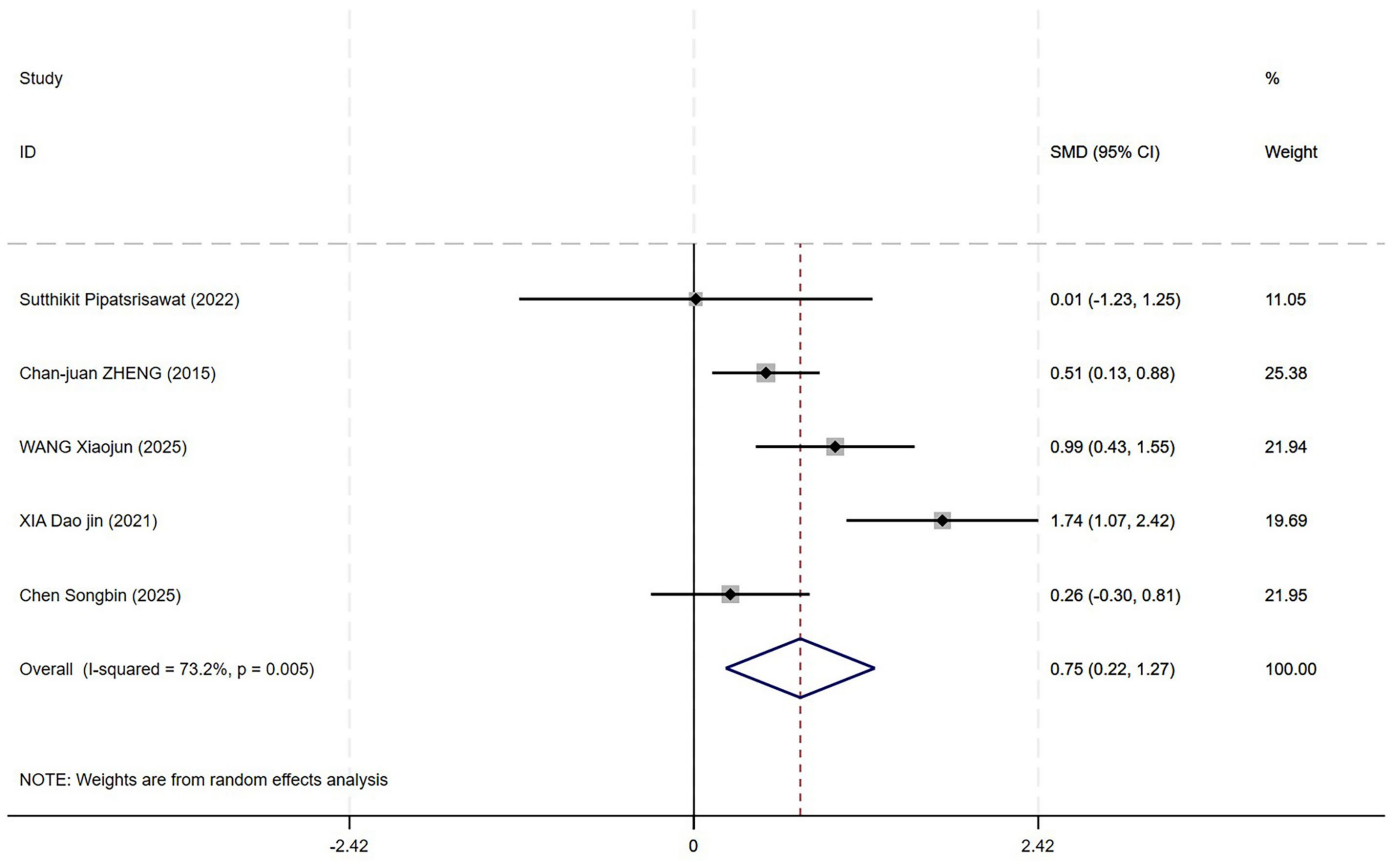
Forest plot of WMFT in subacute stroke. Squares indicate study weight; the diamond represents the overall effect; positive values favor the experimental group.

Five studies (23, 32, 41, 43, 44) (*n* = 276) were included. NINS was associated with a statistically substantial between-group difference in upper limb motor performance based on post-intervention data [SMD = 0.75, 95% CI (0.22, 1.27), *p* < 0.05], with substantial heterogeneity (*I*^2^ = 73.2%) (Figure 6).

#### 3.4.6 MAS

**Figure 7:**
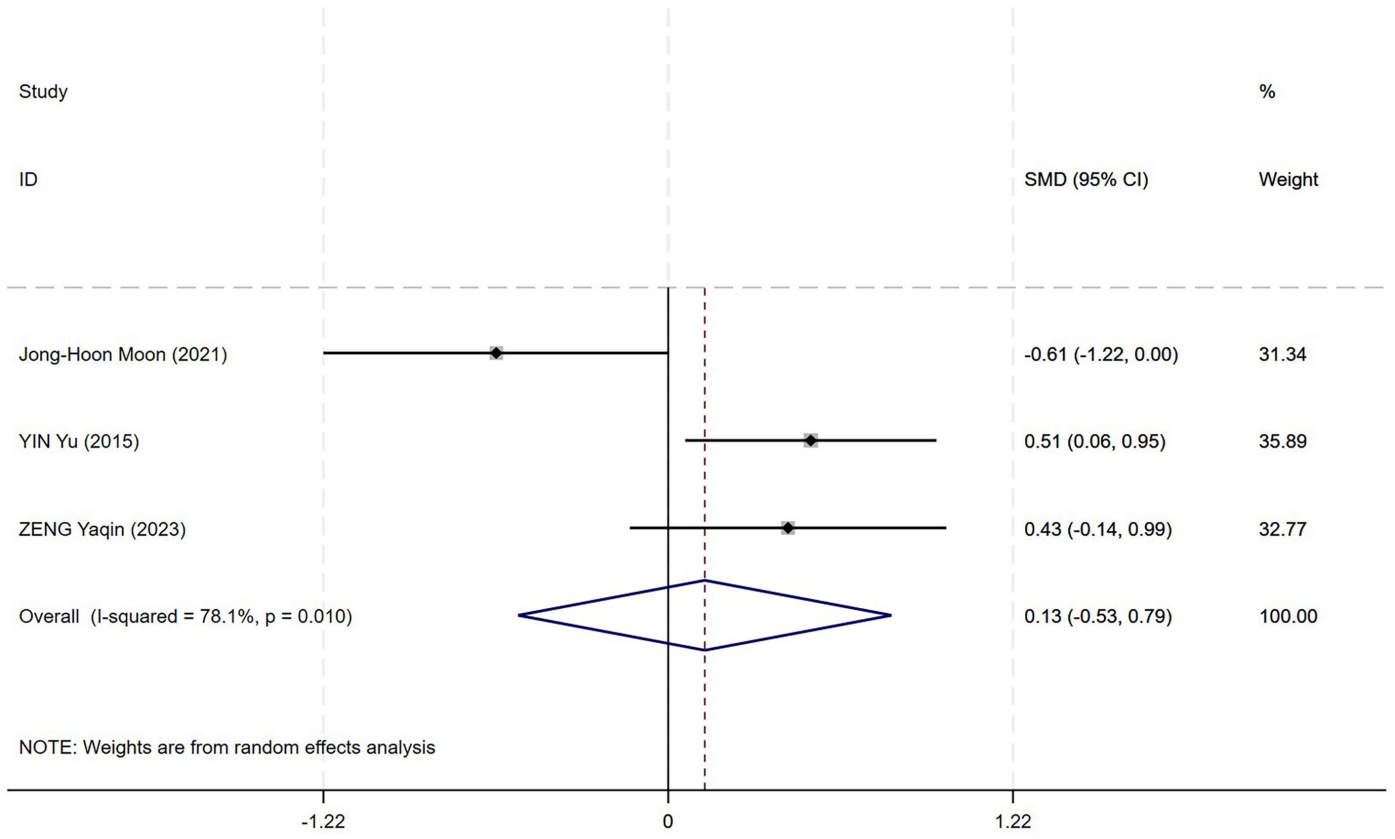
Forest plot of MAS in subacute stroke. Squares indicate study weight; the diamond represents the overall effect; lower values indicate improvement in the experimental group.

Three studies (24, 38, 39) (*n* = 173) were included. No statistically substantial between-group difference in spasticity was observed based on post-intervention data [SMD = 0.13, 95% CI (−0.53, 0.79), *p* = 0.701], with substantial heterogeneity (*I*^2^ = 78.1%). Given the direction of the MAS scale, higher scores indicate greater spasticity (Figure 7).

### 3.5 Sensitivity analysis

Sensitivity analyses showed that the overall direction of effects remained largely consistent after sequential exclusion of individual studies, suggesting that no single study disproportionately influenced the pooled estimates. However, given the substantial heterogeneity and the limited number of studies for certain outcomes, these findings should be interpreted with caution. Detailed results are presented in Supplementary Figures S3–S8.

### 3.6 Subgroup and meta-regression analyses

Given the substantial heterogeneity across outcomes and the limited number of studies, subgroup and meta-regression analyses were conducted based on stimulation modalities and recovery stage as exploratory analyses. Accordingly, their findings should be interpreted with caution and were not intended for direct comparisons between different stimulation modalities.

#### 3.6.1 FMA-UE

Based on post-intervention data, statistically substantial between-group differences were observed in the early subacute stage (*n* = 12 studies) [SMD = 0.63, 95% CI (0.19, 1.07), *I*^2^ = 82.0%] and the late subacute stage (*n* = 10 studies) [SMD = 0.56, 95% CI (0.16, 0.96), *I*^2^ = 73.7%], with substantial heterogeneity in both subgroups. Across NINS modalities, statistically substantial between-group differences were observed for tDCS (*n* = 9 studies) [SMD = 0.82, 95% CI (0.49, 1.15), *I*^2^ = 64.4%], taVNS (*n* = 2 studies) [SMD = 0.86, 95% CI (0.45, 1.27), *I*^2^ = 0.0%], and TEAS (*n* = 1 study) [SMD = 1.67, 95% CI (1.00, 2.33)]. Meta-regression did not identify statistically substantial associations between effect size and recovery stage [*β* = 0.08, 95% CI (−0.61, 0.77), *p* = 0.82] or stimulation modalities [*β* = 0.01, 95% CI (−0.17, 0.19), *p* = 0.93]. Meta-regression results are presented in Supplementary Table S2, and subgroup analysis results are shown in Supplementary Figures S9, S10.

#### 3.6.2 Barthel Index

Based on post-intervention data, statistically substantial between-group differences were observed in the early subacute stage (*n* = 9 studies) [SMD = 0.90, 95% CI (0.37, 1.44), *I*^2^ = 87.5%] and the late subacute stage (*n* = 8 studies) [SMD = 0.63, 95% CI (0.15, 1.11), *I*^2^ = 79.8%], with substantial heterogeneity in both subgroups. Across NINS modalities, statistically substantial between-group differences were observed for tDCS (*n* = 7 studies) [SMD = 0.86, 95% CI (0.26, 1.47), *I*^2^ = 86.7%] and TEAS (*n* = 1 study) [SMD = 2.46, 95% CI (1.70, 3.23)]. Meta-regression did not identify statistically substantial associations between effect size and recovery stage [*β* = 0.27, 95% CI (−0.58, 1.13), *p* = 0.51] or stimulation modalities [*β* = 0.05, 95% CI (−0.13, 0.23), *p* = 0.58]. Subgroup analysis results are presented in Supplementary Figures S11, S12.

#### 3.6.3 ARAT

Based on post-intervention data, statistically substantial between-group differences were observed in both the early subacute stage (*n* = 6 studies) [SMD = 0.65, 95% CI (0.12, 1.17), *I*^2^ = 80.8%] and the late subacute stage (*n* = 7 studies) [SMD = 0.70, 95% CI (0.47, 0.94), *I*^2^ = 0.0%]. Across NINS modalities, statistically substantial between-group differences were observed for tDCS (*n* = 7 studies) [SMD = 0.61, 95% CI (0.25, 0.96), *I*^2^ = 62.3%], taVNS (*n* = 2 studies) [SMD = 0.94, 95% CI (0.47, 1.42), *I*^2^ = 22.2%], TEAS (*n* = 1 study) [SMD = 1.23, 95% CI (0.60, 1.85)], and iTBS (*n* = 1 study) [SMD = 0.81, 95% CI (0.23, 1.39)]. Meta-regression did not identify statistically substantial associations between effect size and recovery stage [*β* = −0.06, 95% CI (−0.69, 0.58), *p* = 0.85] or stimulation modalities [*β* = 0.04, 95% CI (−0.09, 0.18), *p* = 0.50]. Subgroup analysis results are presented in Supplementary Figures S13, S14.

#### 3.6.4 BBT

Based on post-intervention data, no statistically substantial between-group differences were observed in either the early subacute stage (*n* = 1 study) [SMD = −0.25, 95% CI (−1.03, 0.52)] or the late subacute stage (*n* = 4 studies) [SMD = 0.24, 95% CI (−0.44, 0.92), *I*^2^ = 70.1%], with substantial heterogeneity in the latter subgroup. Across NINS modalities, a statistically substantial between-group difference was observed for tDCS (*n* = 1 study) [SMD = 0.95, 95% CI (0.38, 1.53)]. Meta-regression did not identify statistically substantial associations between effect size and recovery stage [*β* = −0.50, 95% CI (−2.96, 1.97), *p* = 0.57] or stimulation modalities [*β* = −1.03, 95% CI (−2.35, 0.28), *p* = 0.09]. Subgroup analysis results are presented in Supplementary Figures S15, S16.

#### 3.6.5 WMFT

Based on post-intervention data, no statistically substantial between-group differences were observed in either the early subacute stage (*n* = 3 studies) [SMD = 0.82, 95% CI (−0.14, 1.78), *I*^2^ = 82.4%] or the late subacute stage (*n* = 2 studies) [SMD = 0.62, 95% CI (−0.10, 1.34), *I*^2^ = 70.2%], with substantial heterogeneity in both subgroups. Across NINS modalities, statistically substantial between-group differences were observed for rTMS (*n* = 2 studies) [SMD = 0.46, 95% CI (0.10, 0.82), *I*^2^ = 0.0%], tDCS (*n* = 1 study) [SMD = 0.99, 95% CI (0.43, 1.55)], and TEAS (*n* = 1 study) [SMD = 1.74, 95% CI (1.07, 2.42)]. Meta-regression did not identify statistically substantial associations between effect size and recovery stage [*β* = 0.20, 95% CI (−1.96, 2.36), *p* = 0.78] or stimulation modalities [*β* = 0.19, 95% CI (−0.19, 0.56), *p* = 0.22]. Subgroup analysis results are presented in Supplementary Figures S17, S18.

#### 3.6.6 MAS

Based on post-intervention data, only studies from the early subacute stage (*n* = 3 studies) were available. The pooled analysis showed no statistically substantial between-group difference in spasticity [SMD = 0.13, 95% CI (−0.53, 0.79), *I*^2^ = 78.1%], with substantial heterogeneity. In modality-based subgroup analysis, a statistically substantial between-group difference was observed in the tDCS subgroup (*n* = 2 studies) [SMD = 0.47, 95% CI (0.13, 0.82), *I*^2^ = 0.0%]. Given the direction of the MAS scale, a positive SMD indicates higher spasticity scores. Meta-regression did not identify statistically substantial associations between effect size and stimulation modalities [*β* = −0.15, 95% CI (−0.81, 0.50), *p* = 0.20]. Subgroup analysis results are presented in Supplementary Figures S19, S20.

### 3.7 Publication bias

Egger’s test did not indicate evidence of substantial publication bias (*p* > 0. 05). However, given the limited number of studies for some outcomes, the statistical power of the test was limited, and bias cannot be excluded. Funnel plots appeared relatively symmetrical for FMA-UE, whereas some asymmetry was observed for other outcomes, suggesting a potential risk of bias. Funnel plots are shown in Supplementary Figures S21–S26, and Egger’s test results are presented in Supplementary Figures S27–S32.

## 4 Discussion

This meta-analysis of RCTs indicates that non-invasive neural stimulation (NINS), as an adjunct to conventional rehabilitation or sham stimulation, is associated with modest improvements in upper limb function and activities of daily living in patients with subacute stroke. However, effects were not consistent across outcomes, with no substantial benefits observed for measures such as BBT and MAS. In particular, the absence of substantial improvement in spasticity contrasts with the results reported by Wang et al. (46), who suggested that non-invasive neural stimulation may improve upper limb spasticity after stroke. Overall, these findings are broadly consistent with previous systematic reviews supporting a potential but variable role of NINS in post-stroke rehabilitation (12). Notably, the magnitude of effect and its clinical relevance remain uncertain, particularly given the reliance on standardized mean differences and the inconsistent alignment with established minimal clinically important differences (MCIDs) (21).

Substantial heterogeneity was observed across most outcomes and was not fully explained by subgroup or meta-regression analyses. This heterogeneity likely reflects a combination of clinical and methodological diversity, including variability in stimulation parameters (e.g., intensity, frequency, and duration), patient characteristics (e.g., lesion location and severity), and concurrent rehabilitation protocols (12, 47). Although subgroup analyses suggested potential benefits across early and late subacute stages, which is consistent with heightened neuroplasticity during this recovery window (8, 9), these findings should be interpreted cautiously. Across stimulation modalities, observed effects were based on indirect evidence, and no formal interaction tests were performed; therefore, comparative effectiveness between modalities cannot be inferred.

Several limitations should be acknowledged. First, substantial heterogeneity persisted despite exploratory analyses, limiting the robustness of pooled estimates. Second, the number of studies in certain subgroups was small, reducing statistical power. Third, formal interaction tests between subgroups were not conducted, constraining interpretation of subgroup differences. Fourth, allocation concealment was inadequately reported in many studies, and incomplete reporting of key variables (e.g., stimulation parameters and patient characteristics) introduces potential risk of bias. In addition, safety data were inconsistently reported, precluding quantitative synthesis. The reliance on post-intervention data also limits the assessment of longitudinal recovery trajectories. Finally, the geographic concentration of included studies may affect generalizability.

Compared with previous reviews, this study provides several methodological and clinical contributions. By focusing specifically on the subacute stage, it offers a more targeted evaluation during a critical window of neuroplasticity. The inclusion of a broader range of stimulation modalities enhances the comprehensiveness of the evidence base. In addition, greater emphasis was placed on methodological rigor, including outcome directionality alignment and cautious interpretation of SMDs. Clinically, NINS may be considered a potential adjunct to rehabilitation; however, its effectiveness across modalities and outcomes remains uncertain. Future research should prioritize well-designed, adequately powered trials with standardized reporting, direct comparisons between modalities, and longer follow-up to clarify treatment effects and their clinical relevance.

## 5 Conclusion

In conclusion, non-invasive neural stimulation (NINS), when used as an adjunct to conventional rehabilitation or sham stimulation, may confer modest, context-dependent improvements in upper limb function and activities of daily living in patients with subacute stroke. However, the robustness and clinical relevance of these effects remain uncertain due to substantial between-study heterogeneity and methodological variability, which preclude firm conclusions regarding comparative efficacy across stimulation modalities. Accordingly, the current evidence should be considered exploratory. Future large-scale, multicenter RCTs with standardized intervention protocols and outcome reporting are warranted to clarify the therapeutic value of NINS and optimize its clinical implementation.

## Data Availability

The original contributions presented in the study are included in the article/Supplementary material, further inquiries can be directed to the corresponding author.
