# Supplementary material for: Effects of non-invasive neural stimulation modalities on upper limb function in subacute stroke: a systematic review and meta-analysis
Source: Front Neurol. 2026 May 29;17:1805246. doi: 10.3389/fneur.2026.1805246 (PMC13259675; doi:10.3389/fneur.2026.1805246)
Supplement: Supplementary file 1 [file Data_Sheet_1.docx]

Table S1 Literature search strategy

|  | Search query |
| --- | --- |
| #1： | (((((stroke[MeSH Terms]) ) OR (apoplexy[Title/Abstract])) OR (cerebral hemorrhage[Title/Abstract])) OR (cerebral infarction[Title/Abstract])) OR (brain vascular accident[Title/Abstract]) OR (CVA[Title/Abstract]) OR (Ischemic stroke[Title/Abstract]) OR (Hemorrhagic stroke[Title/Abstract]) |
| #2： | ((((((((electrical stimulation[MeSH Terms]) OR (magnetic stimulation[Title/Abstract])) OR (transcranial electrical stimulation[Title/Abstract])) OR (transcranial magnetic stimulation[Title/Abstract])) OR (transcranial direct current stimulation[Title/Abstract])) OR (repetitive transcranial magnetic stimulation[Title/Abstract])) OR (intermittent theta-burst stimulation[Title/Abstract])) OR (noninvasive brain stimulation[Title/Abstract]) OR (neuromodulation[Title/Abstract]) OR (non-invasive neural stimulation[Title/Abstract]) OR (peripheral nerve stimulation[Title/Abstract]) OR (central nervous system stimulation[Title/Abstract]) |
| #3： | ((upper extremity[MeSH Terms]) OR (upper limb function[Title/Abstract])) OR (upper limb motor function[Title/Abstract]) OR (limb motor impairment[Title/Abstract]) OR (upper limb disablity[Title/Abstract]) |
| #4： | #1 AND #2 AND #3 |

Table S2 Meta-regression results

|  | variables | coefficient | 95%Cl | Std.Err | P value |
| --- | --- | --- | --- | --- | --- |
| FUMA | time | 0.08 | (-0.61,0.77) | 0.33 | 0.82 |
|  | cons | 0.48 | (-0.64,1.59) | 0.54 | 0.39 |
|  | type | 0.01 | (-0.17,0.19) | 0.09 | 0.93 |
|  | cons | 0.58 | (0.00,1.15) | 0.28 | 0.05 |
| Barthel Index | time | 0.27 | (-0.58,1.13) | 0.40 | 0.51 |
|  | cons | 0.36 | (-1.02,1.73) | 0.65 | 0.59 |
|  | type | 0.05 | (-0.13,0.23) | 0.09 | 0.58 |
|  | cons | 0.63 | (-0.07,1.32) | 0.33 | 0.07 |
| ARAT | time | -0.06 | (-0.69,0.58) | 0.29 | 0.85 |
|  | cons | 0.75 | (-0.24,1.74) | 0.45 | 0.12 |
|  | type | 0.04 | (-0.09,0.18) | 0.06 | 0.50 |
|  | cons | 0.55 | (0.05,1.04) | 0.22 | 0.03 |
| BBT | time | -0.50 | (-2.96,1.97) | 0.77 | 0.57 |
|  | cons | 0.74 | (-2.37,3.85) | 0.98 | 0.51 |
|  | type | -1.03 | (-2.35,0.28) | 0.41 | 0.09 |
|  | cons | 1.99 | (-0.32,4.29) | 0.72 | 0.07 |
| WMFT | time | 0.20 | (-1.96,2.36) | 0.68 | 0.78 |
|  | cons | 0.42 | (-3.14,3.97) | 1.12 | 0.73 |
|  | type | 0.19 | (-0.19,0.56) | 0.12 | 0.22 |
|  | cons | 0.20 | (-1.18,1.57) | 0.43 | 0.68 |
| MAS | time | NA | NA | NA | NA |
|  | cons | 0.13 | (-1.40,1.65) | 0.35 | 0.75 |
|  | type | -0.15 | (-0.81,0.50) | 0.05 | 0.20 |
|  | cons | 0.63 | (-2.02,3.28) | 0.21 | 0.20 |


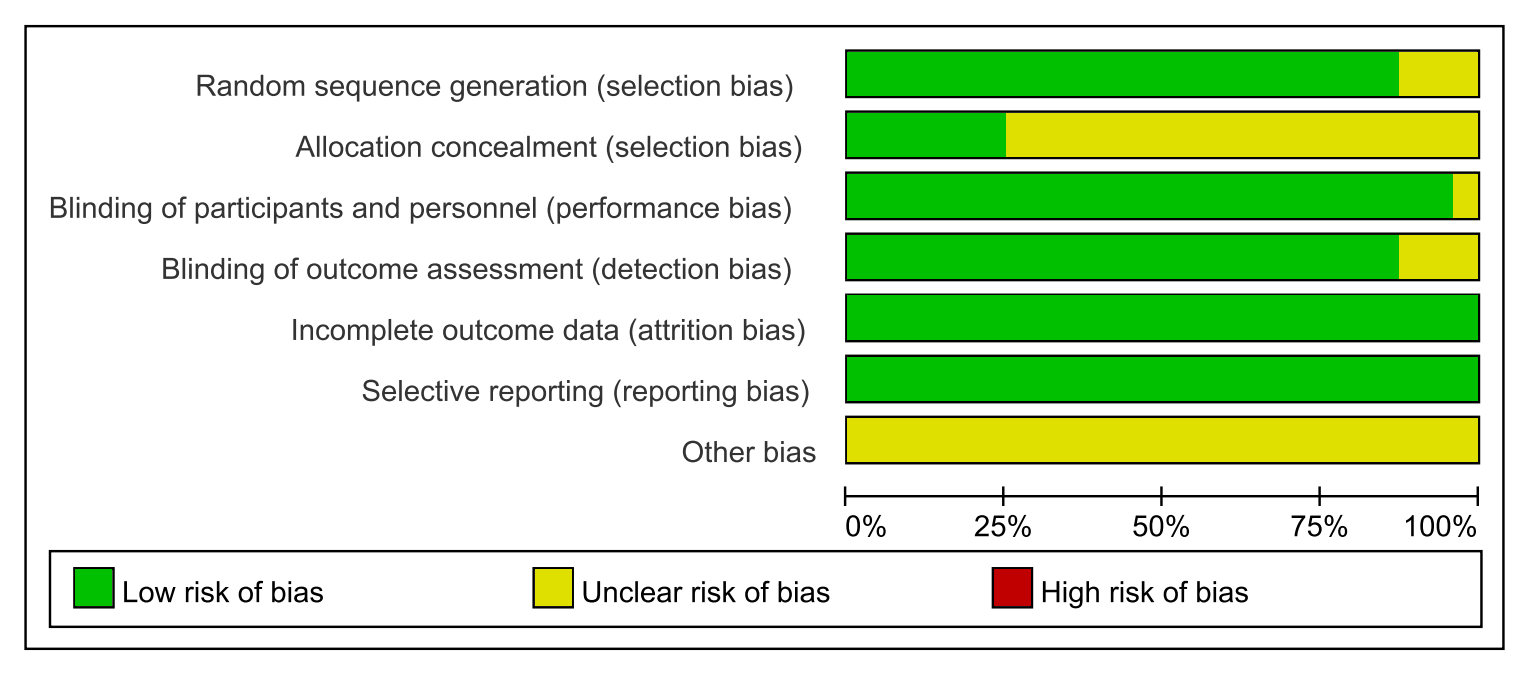


Figure S1 Risk of bias graph.


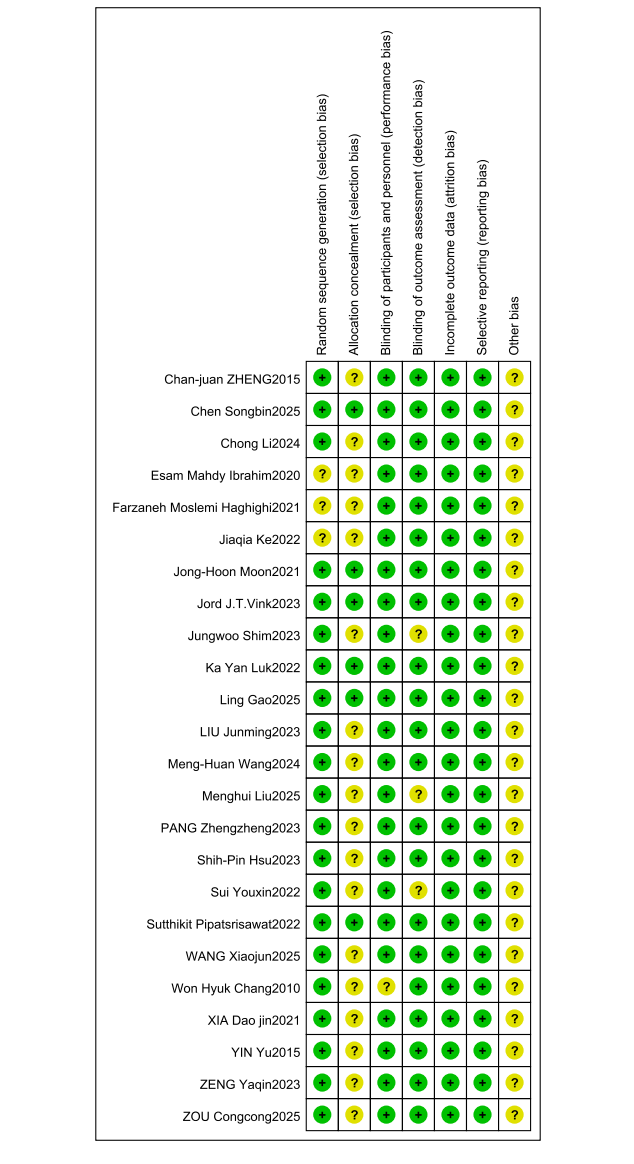


Figure S2 Risk of bias summary.


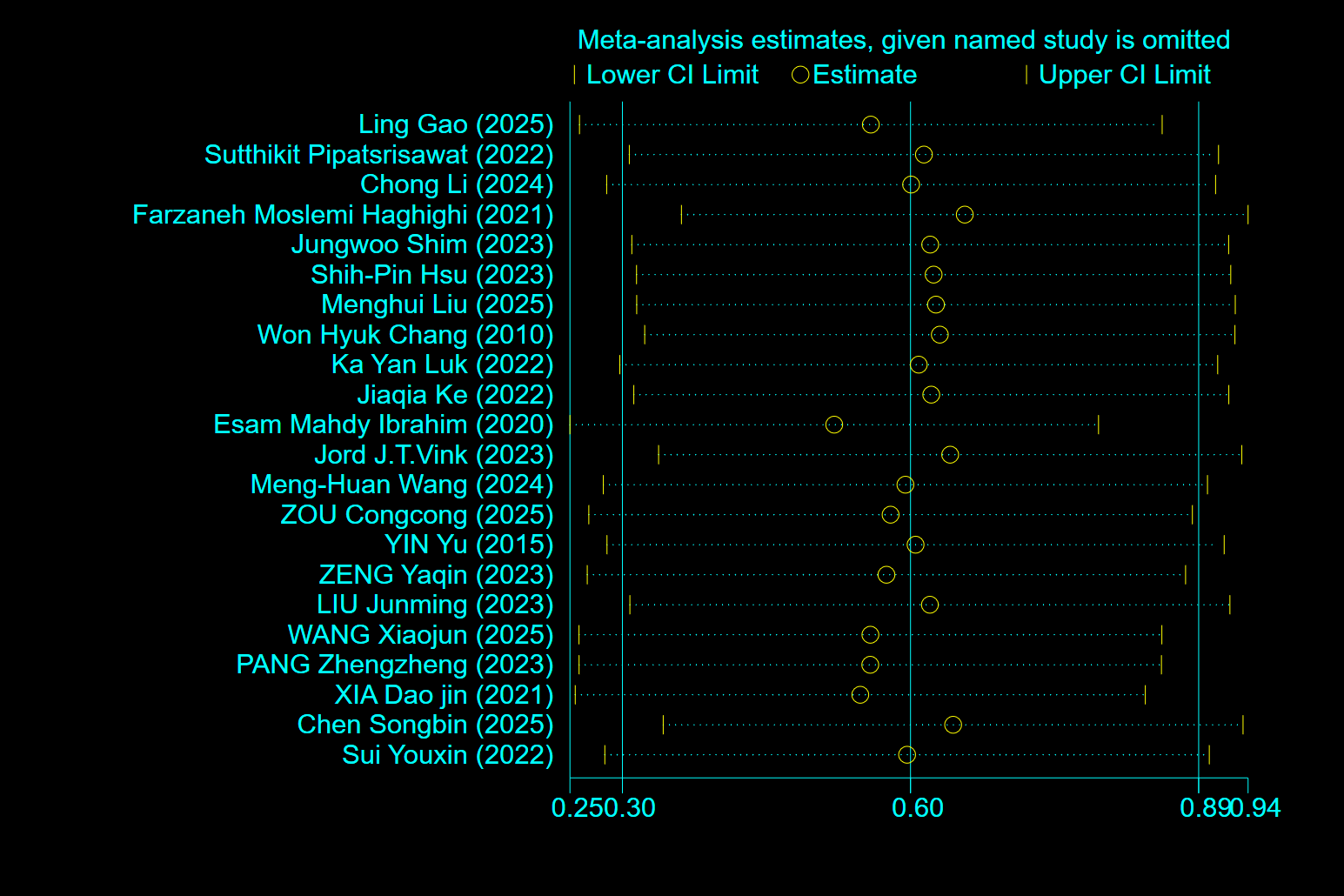


Figure S3 Sensitivity analysis of FMA-UE


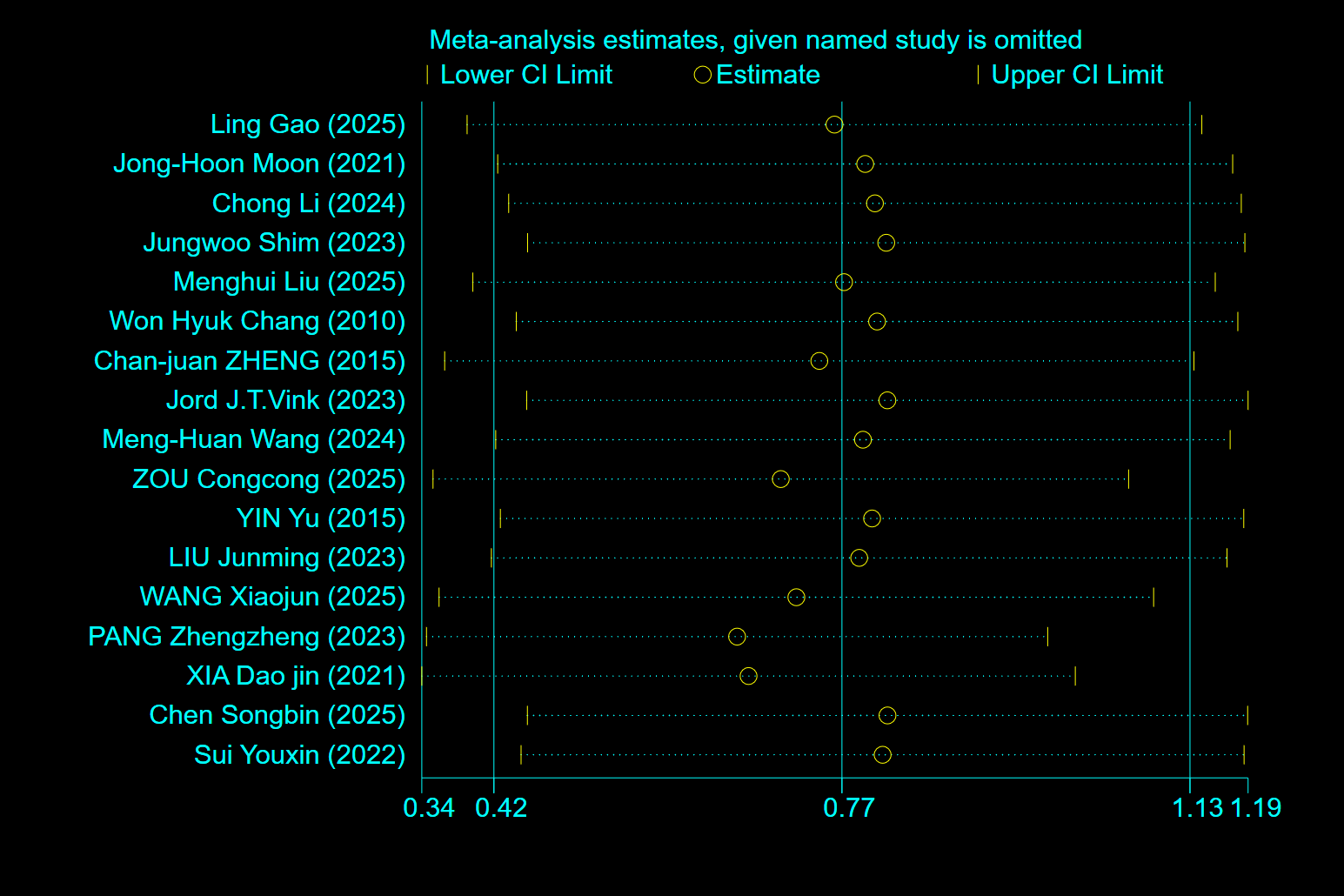


Figure S4 Sensitivity analysis of Barthel Index


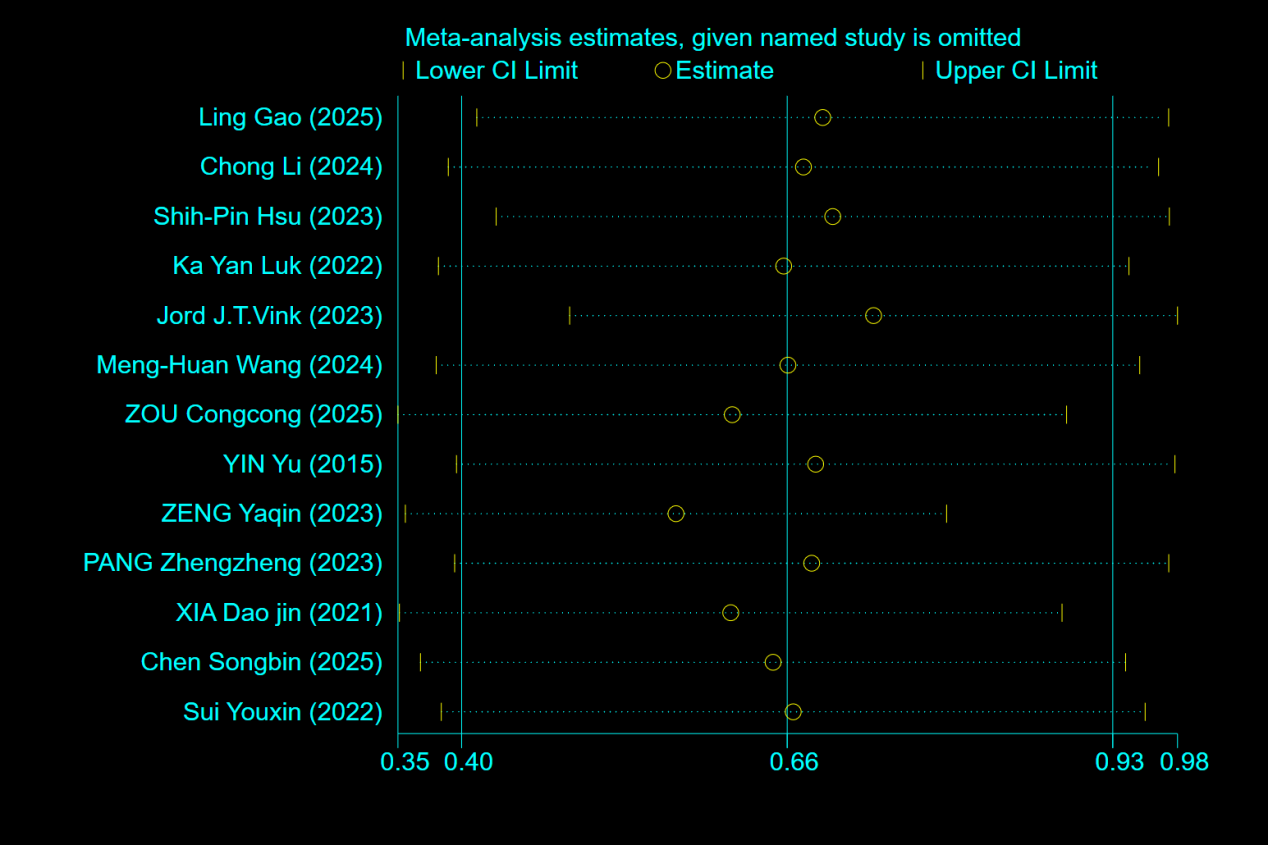


Figure S5 Sensitivity analysis of ARAT


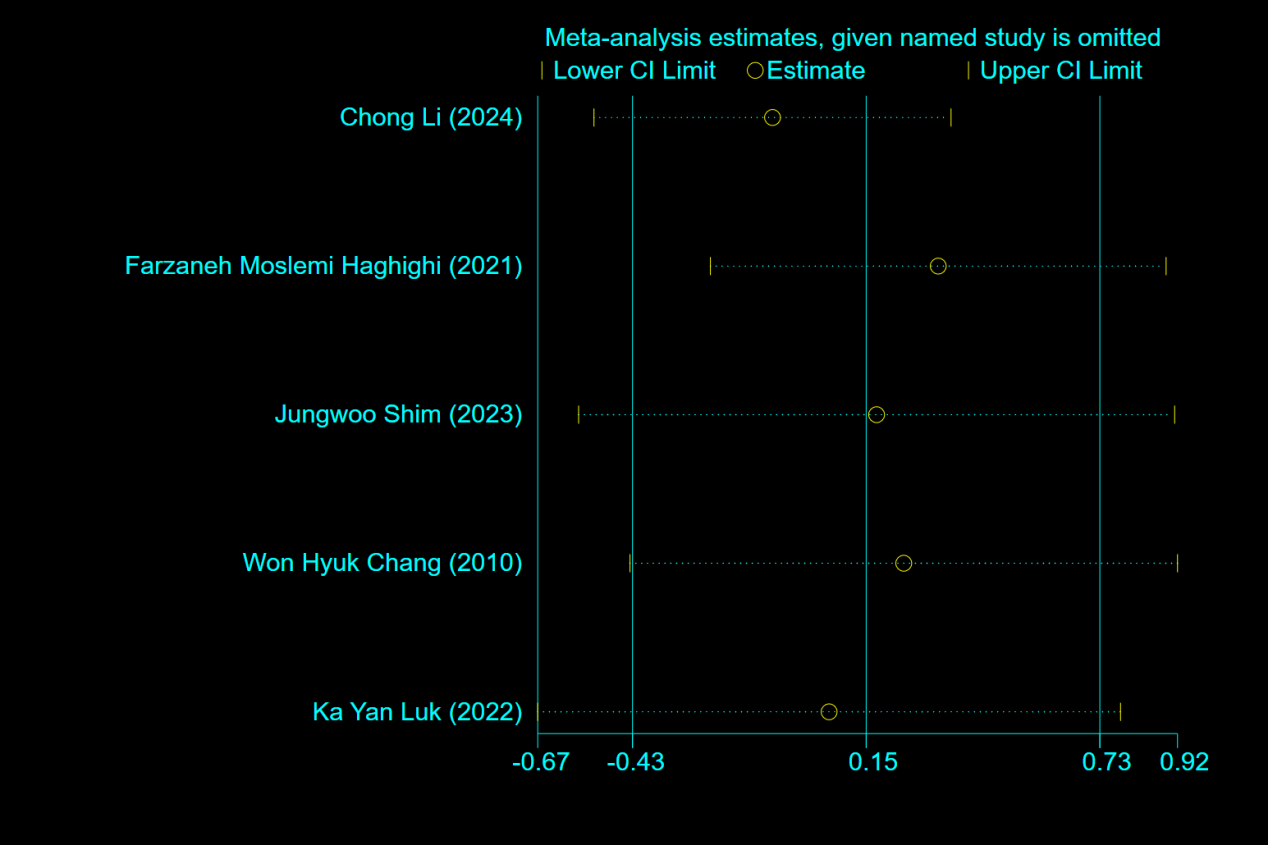


Figure S6 Sensitivity analysis of BBT


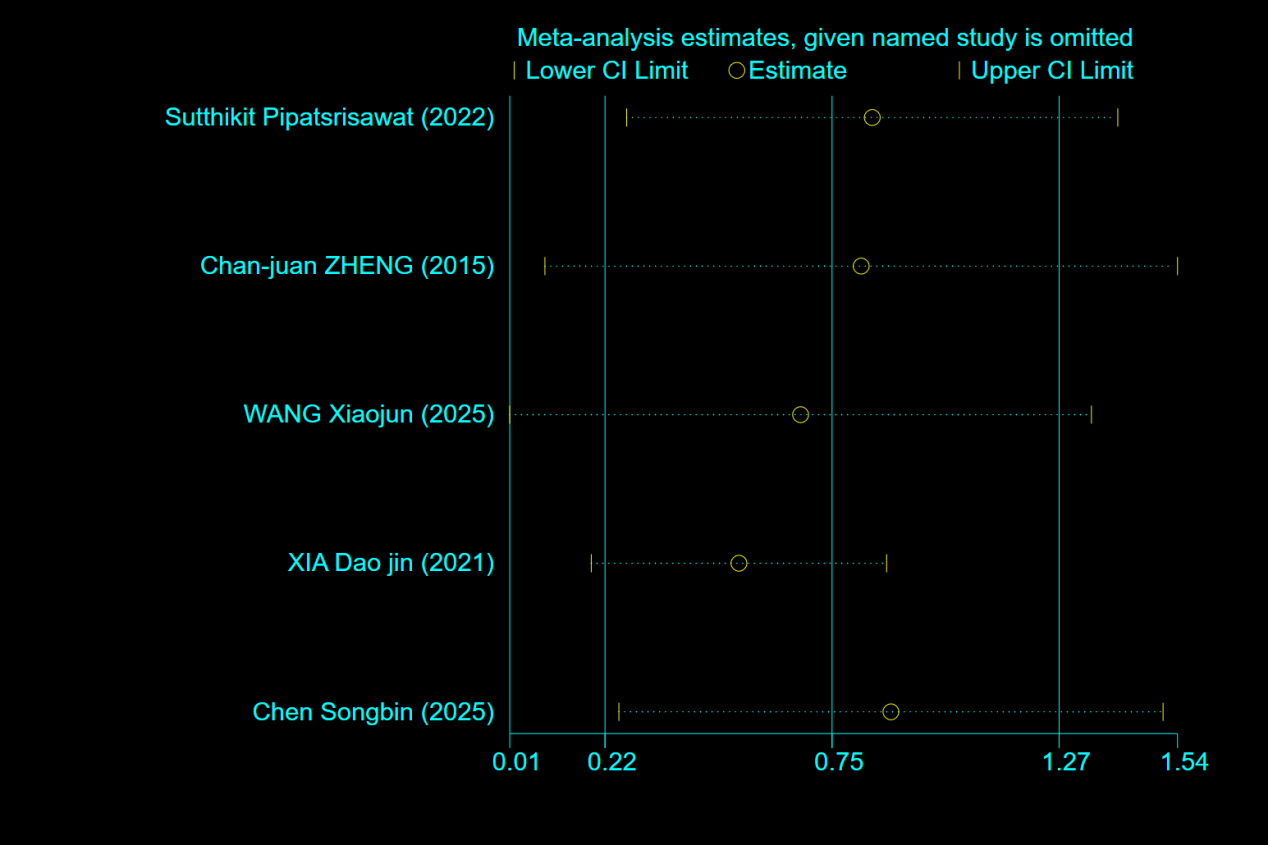


Figure S7 Sensitivity analysis of WMFT


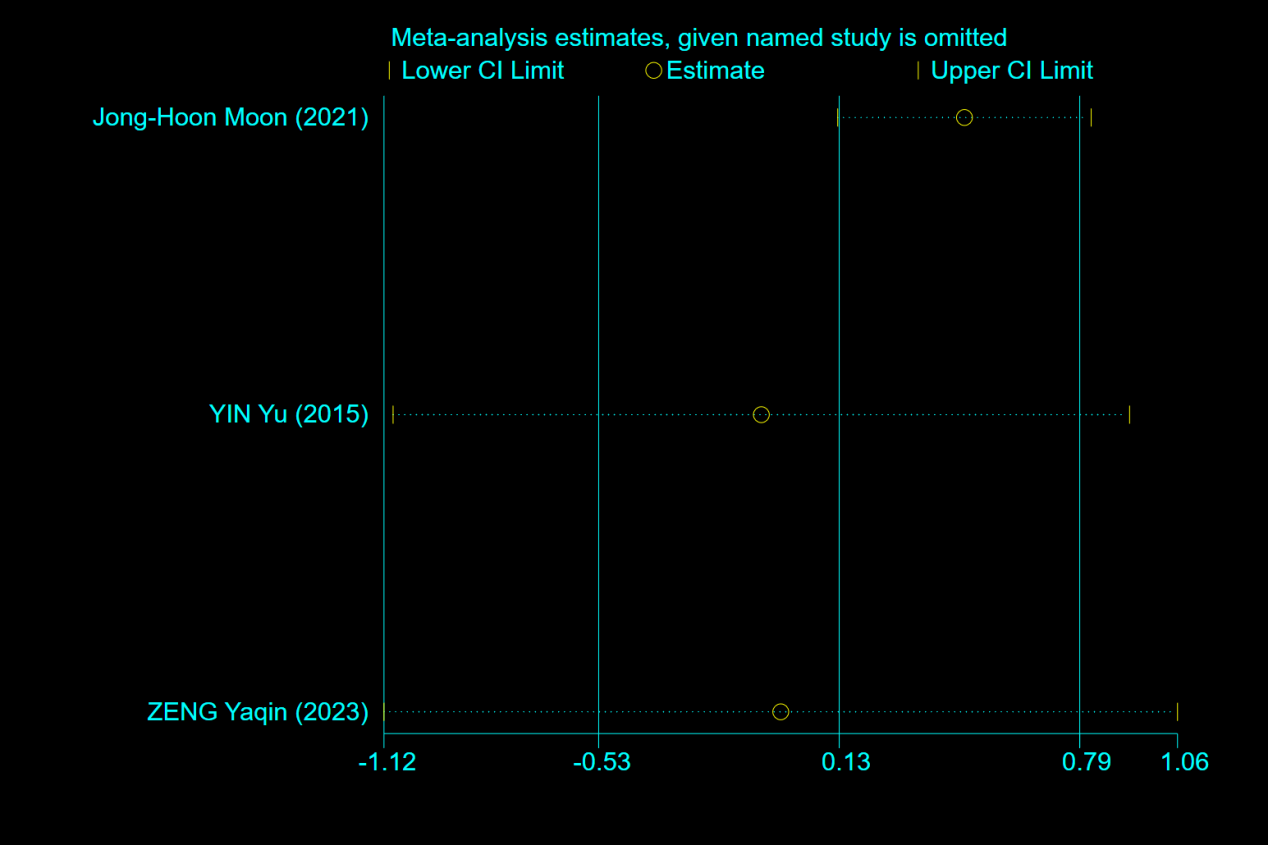


Figure S8 Sensitivity analysis of MAS


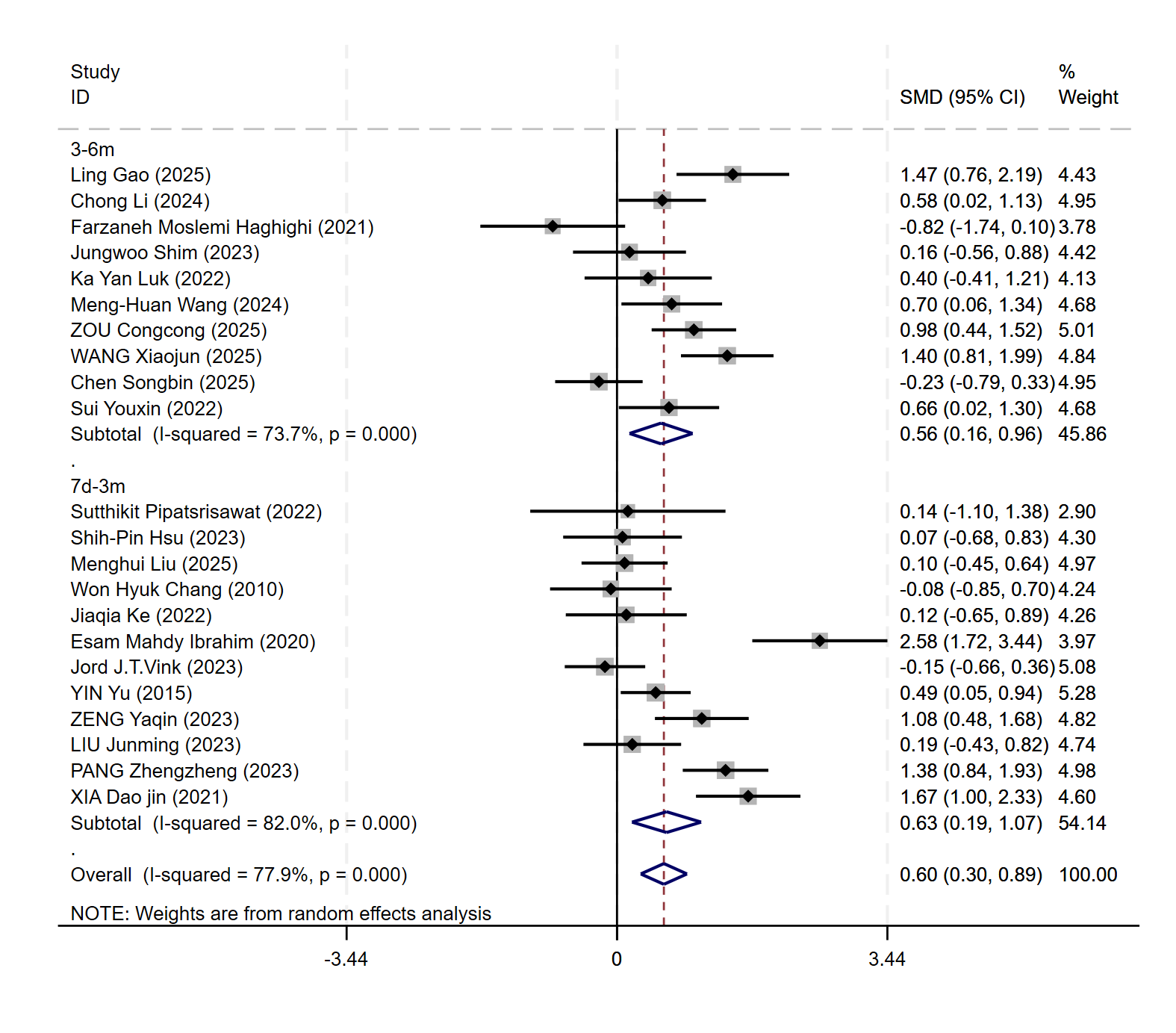


Figure S9.Subgroup Analysis of FMA-UE by Follow-up Time


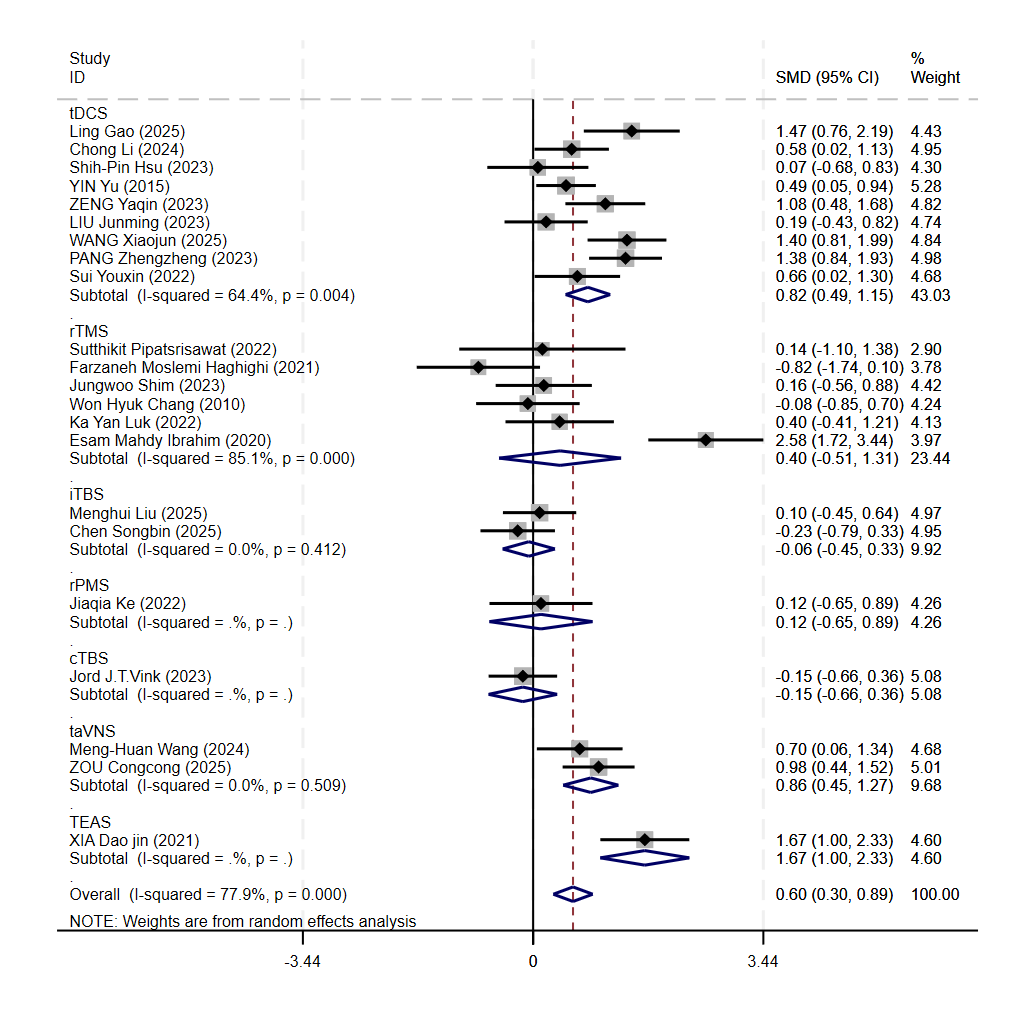


Figure S10.Subgroup Analysis of FMA-UE by Follow-up Type


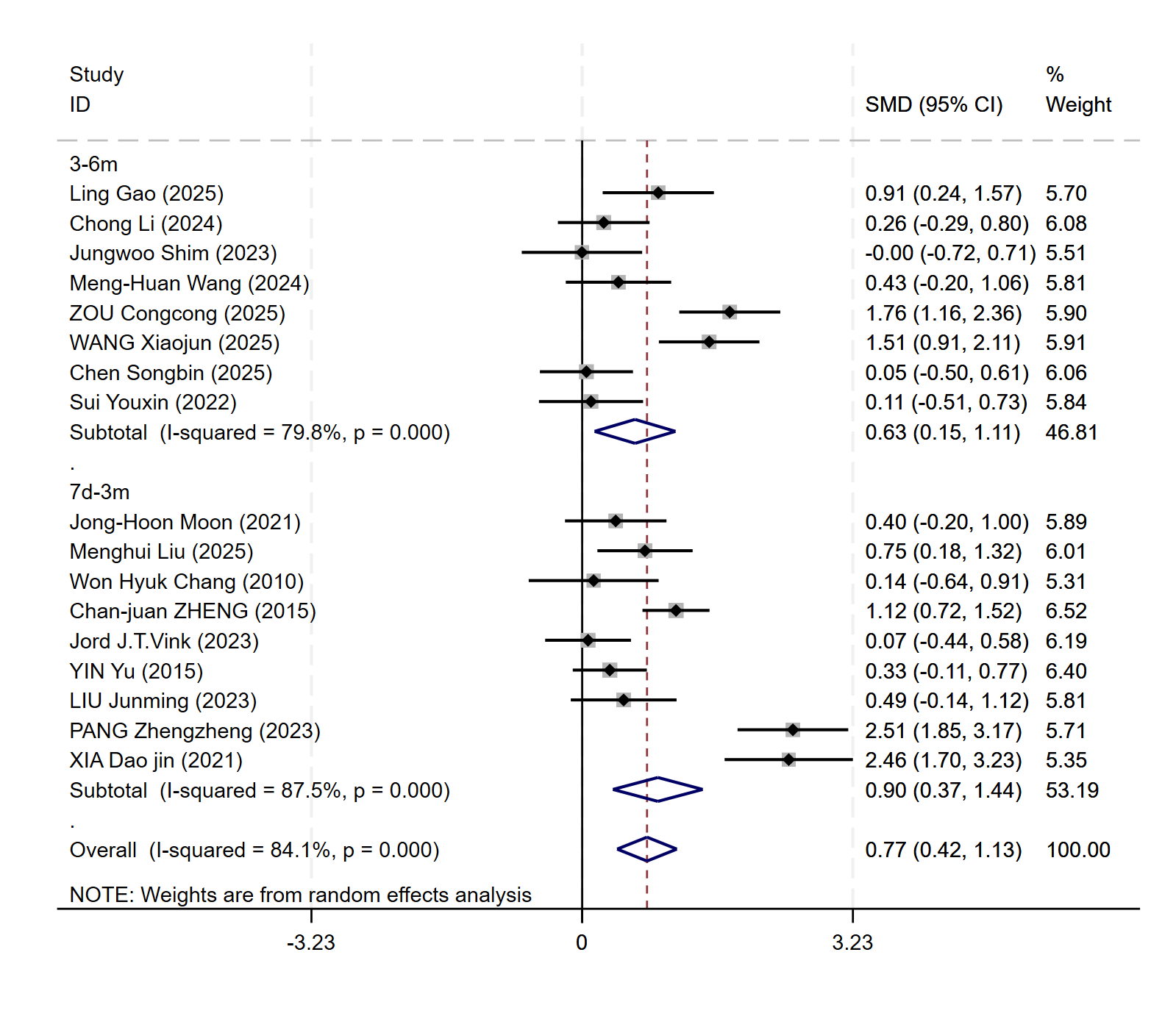


Figure S11.Subgroup Analysis of Barthel Index by Follow-up Time


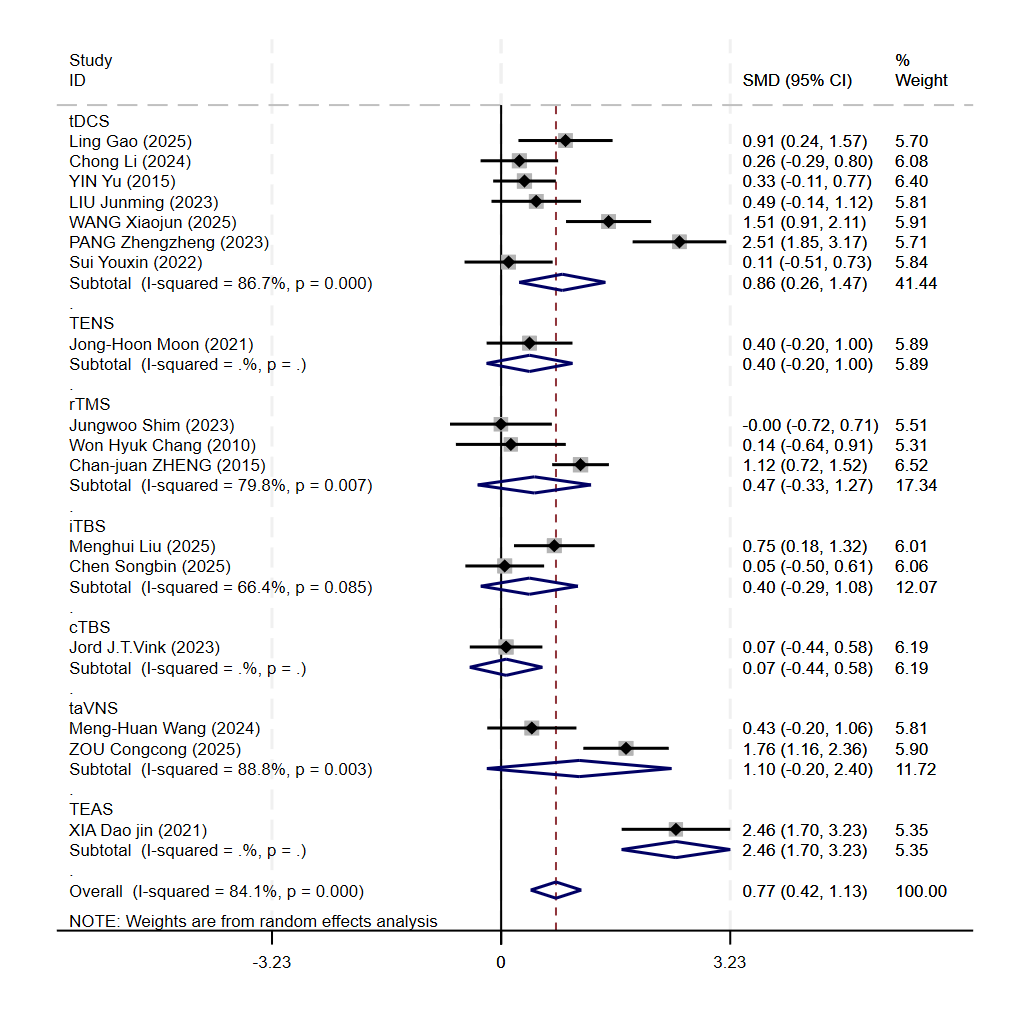


Figure S12.Subgroup Analysis of Barthel Index by Follow-up Type


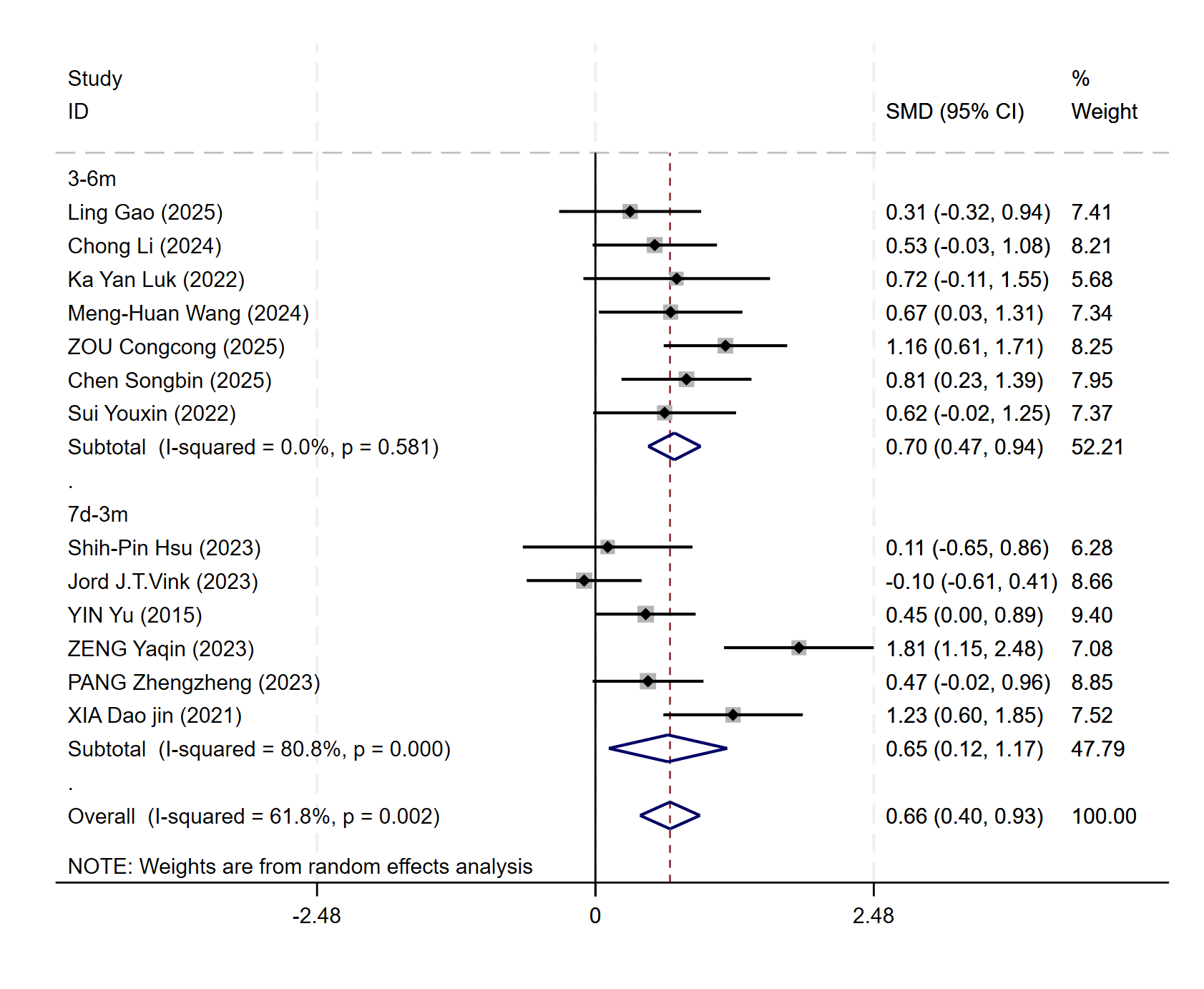


Figure S13.Subgroup Analysis of ARAT by Follow-up Time


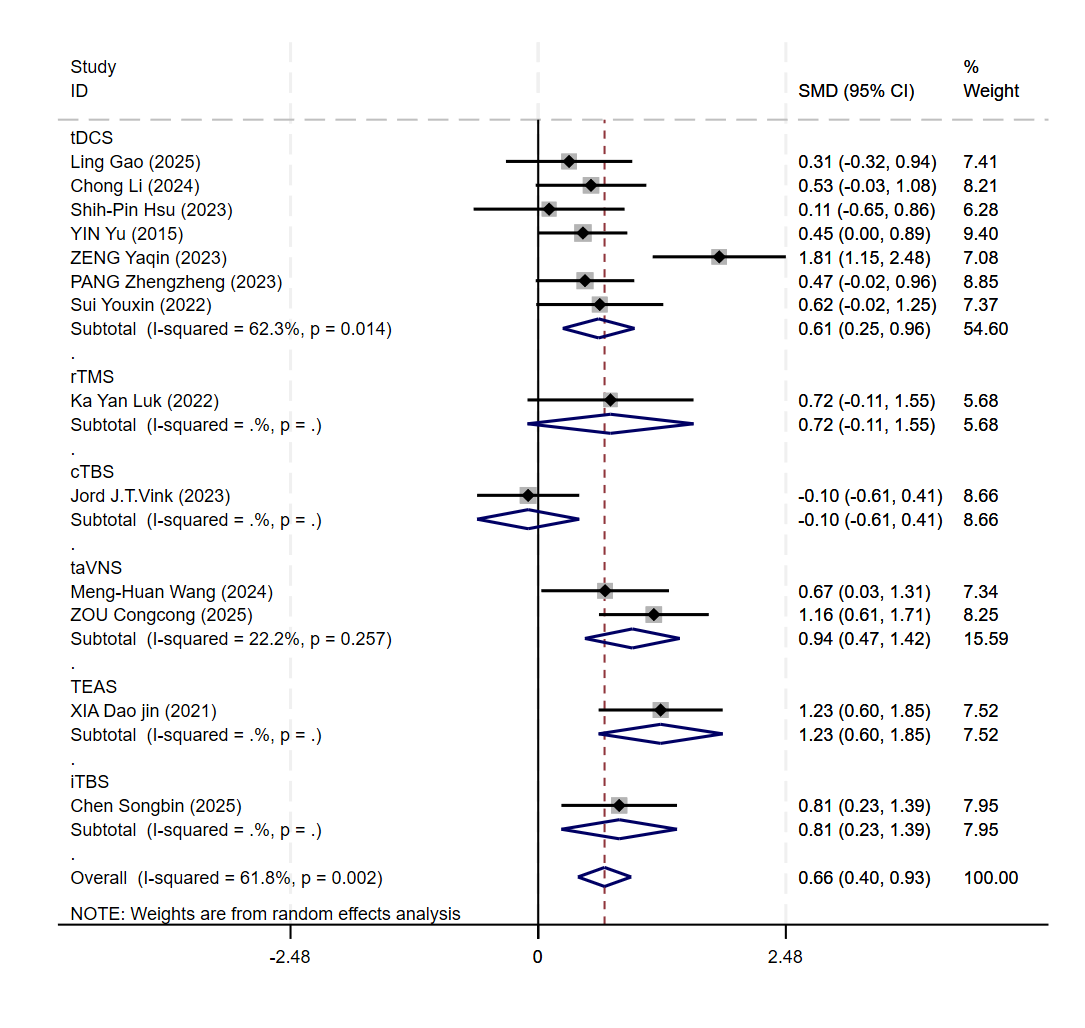


Figure S14.Subgroup Analysis of ARAT by Follow-up Type


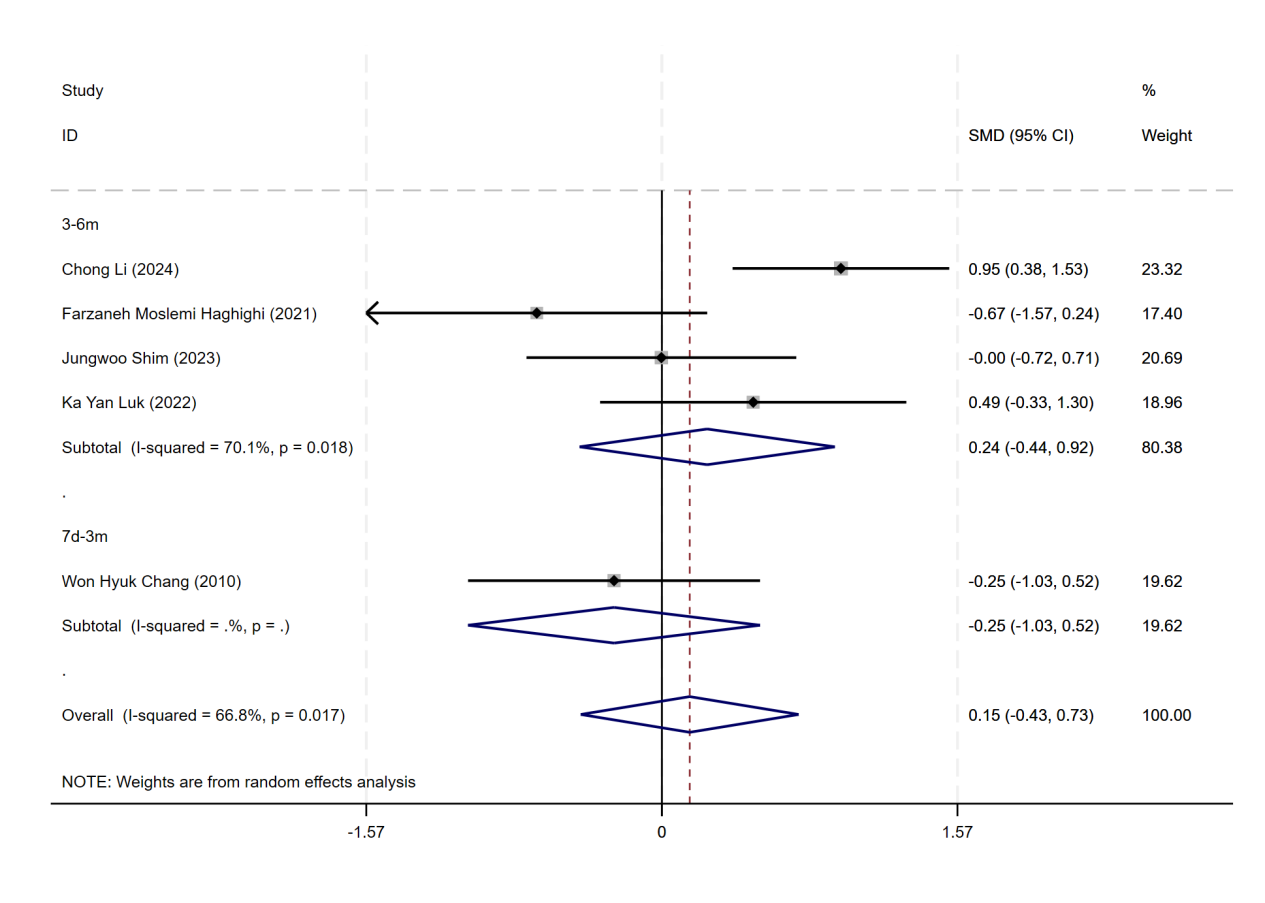


Figure S15.Subgroup Analysis of BBT by Follow-up Time


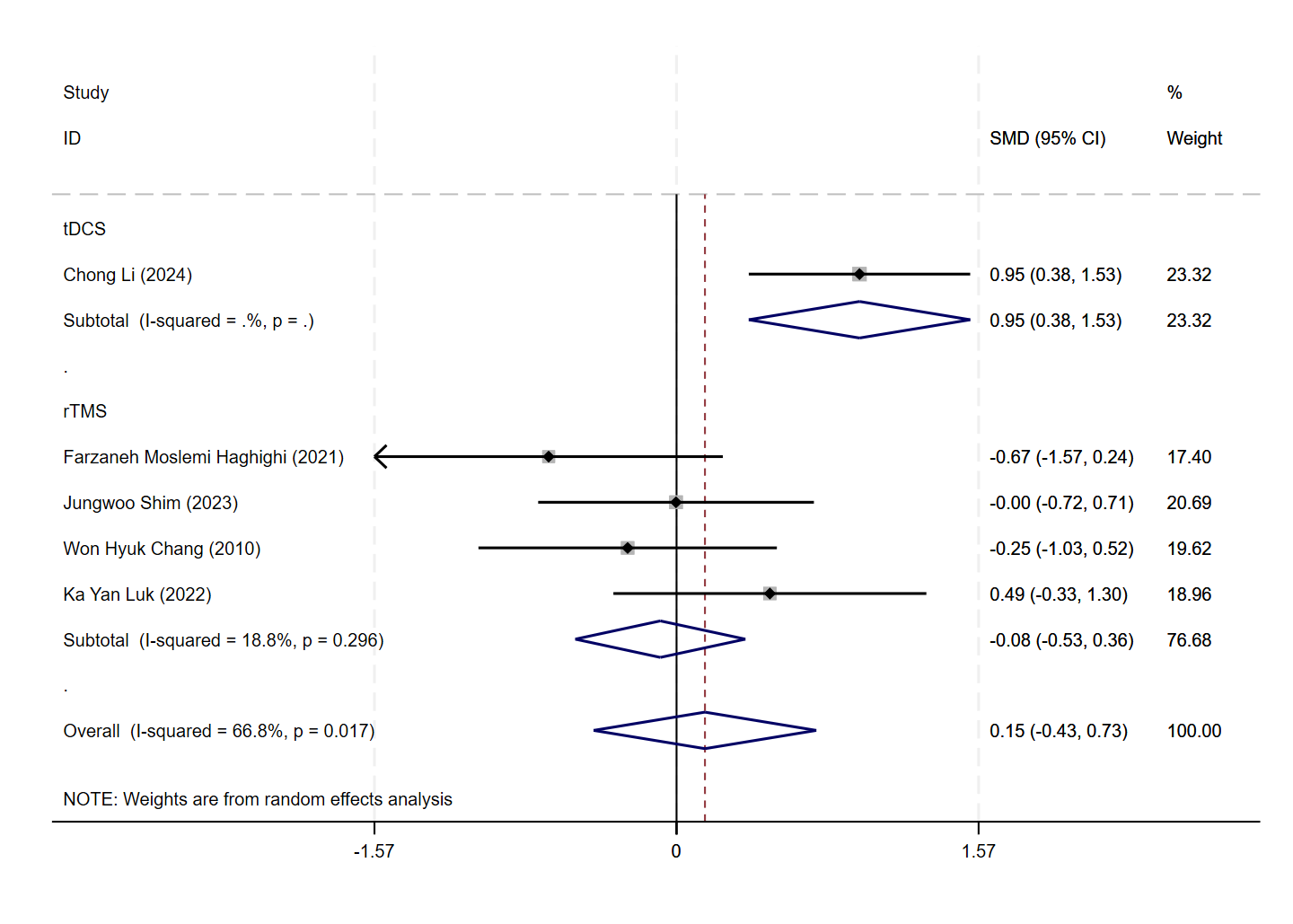


Figure S16.Subgroup Analysis of BBT by Follow-up Type


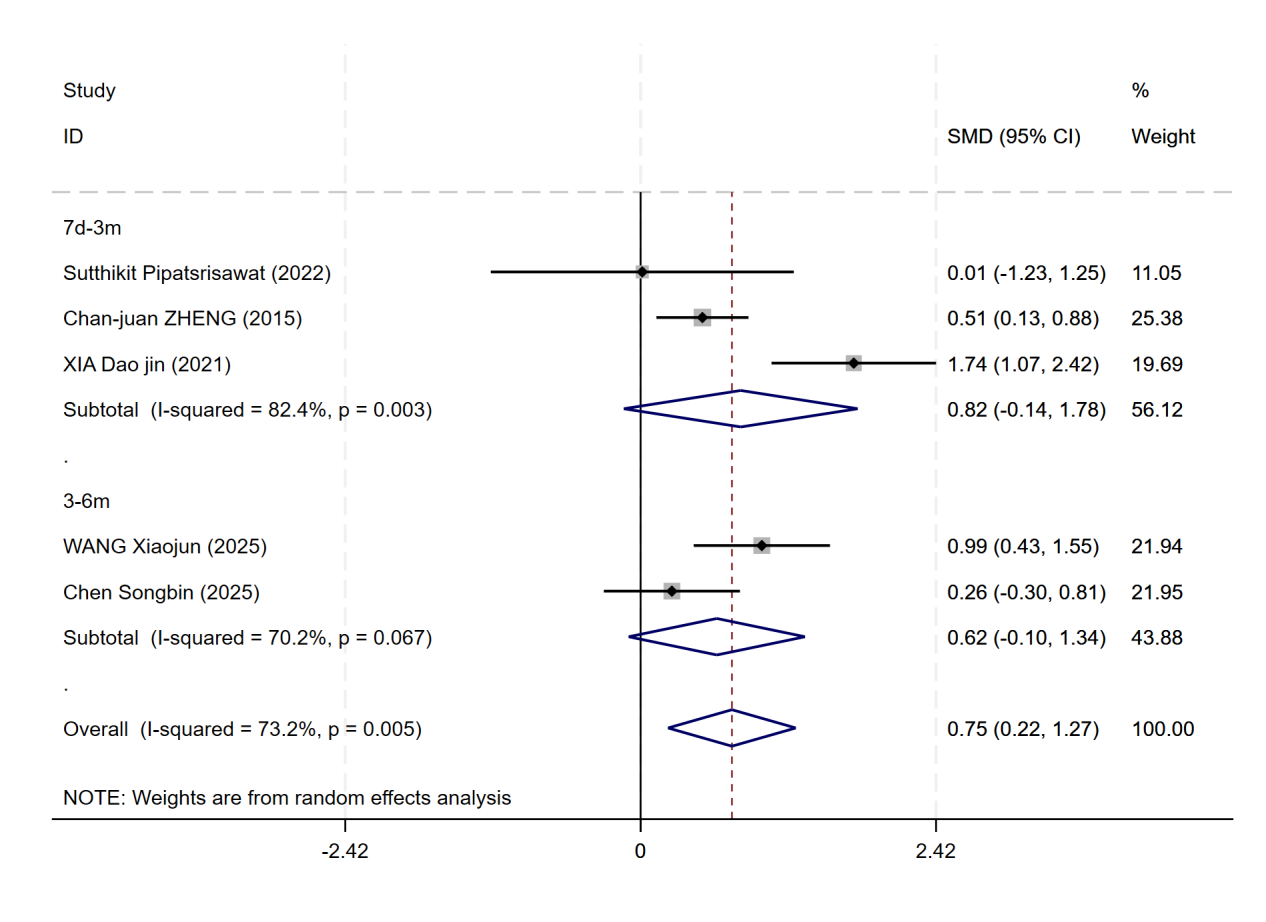


Figure S17.Subgroup Analysis of WMFT by Follow-up Time


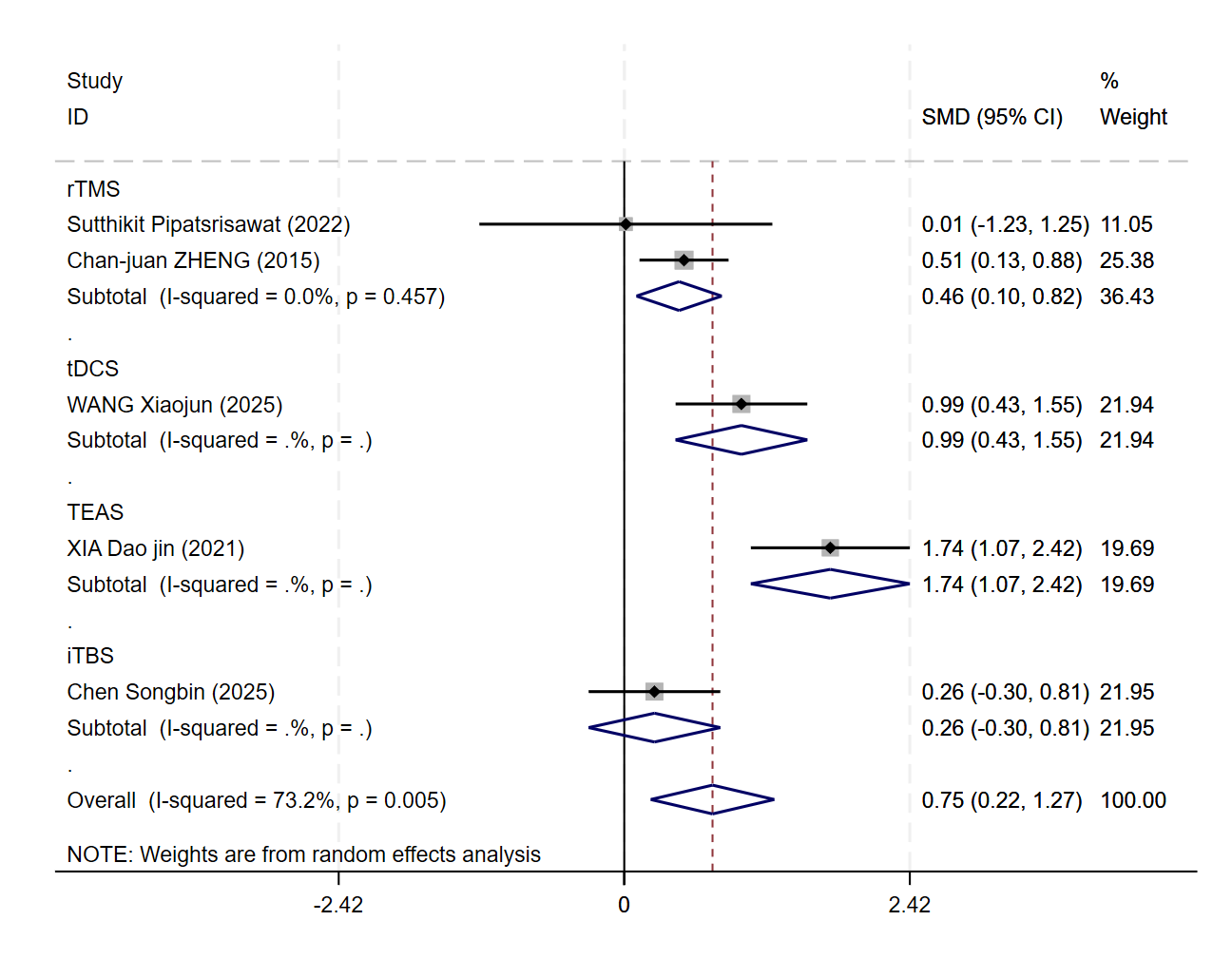


Figure S18.Subgroup Analysis of WMFT by Follow-up Type


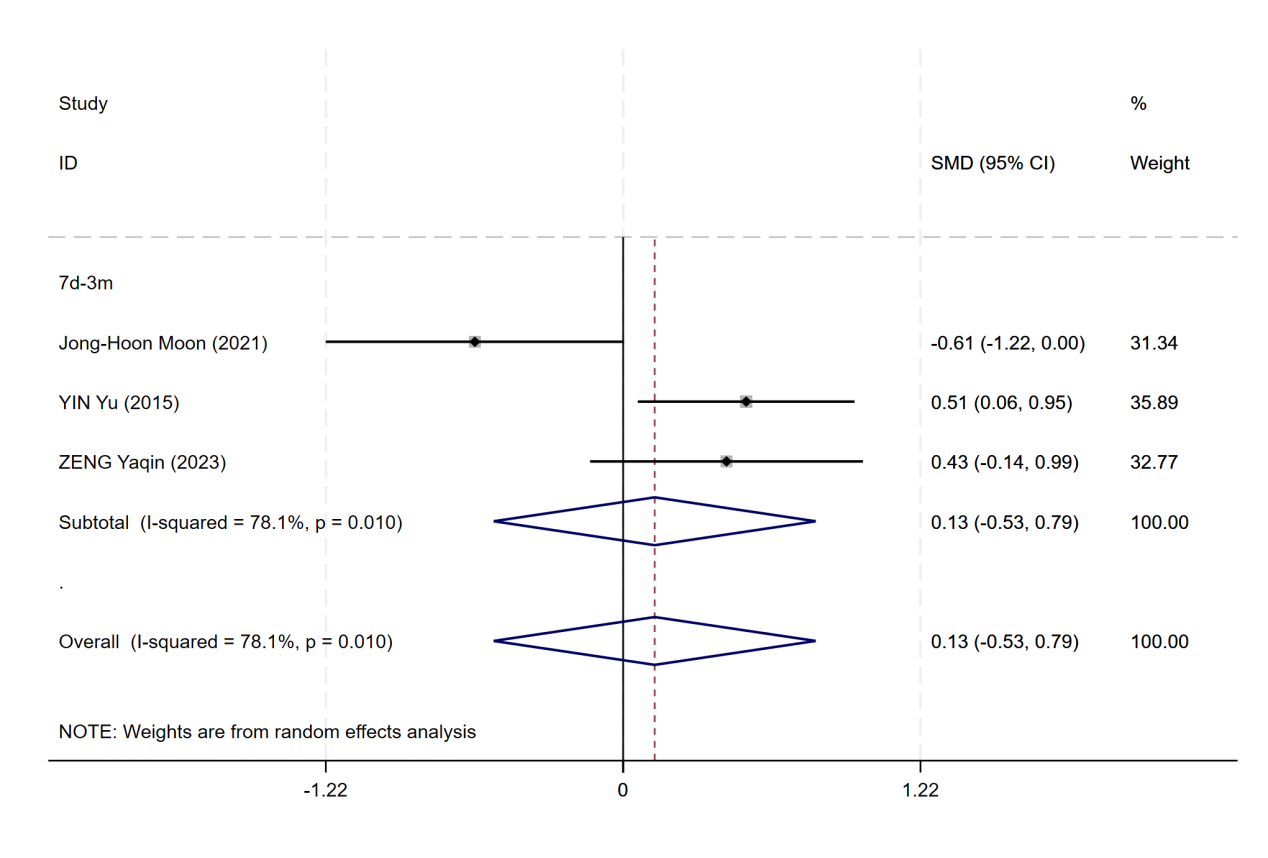


Figure S19.Subgroup Analysis of MAS by Follow-up Time


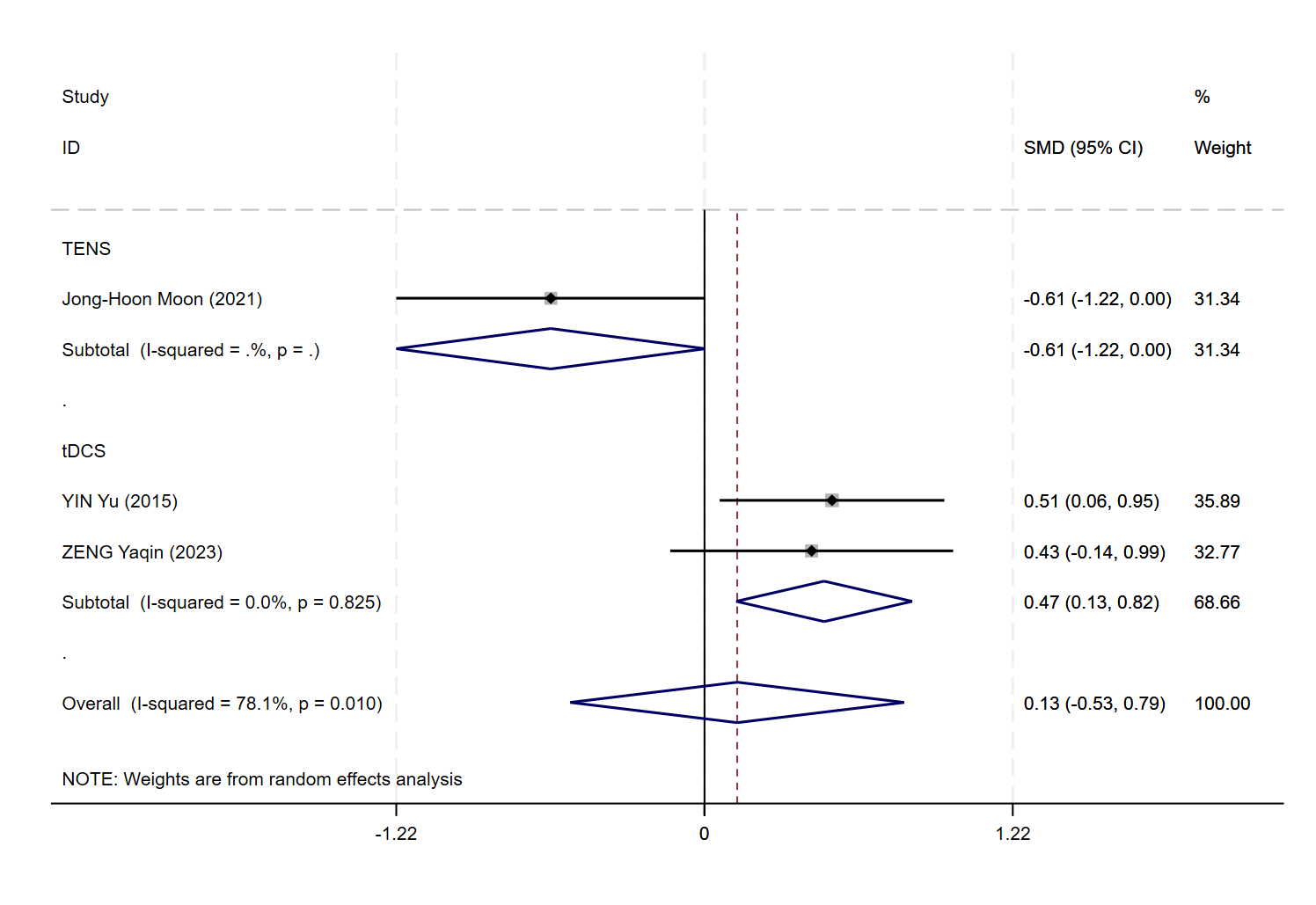


Figure S20.Subgroup Analysis of MAS by Follow-up Type


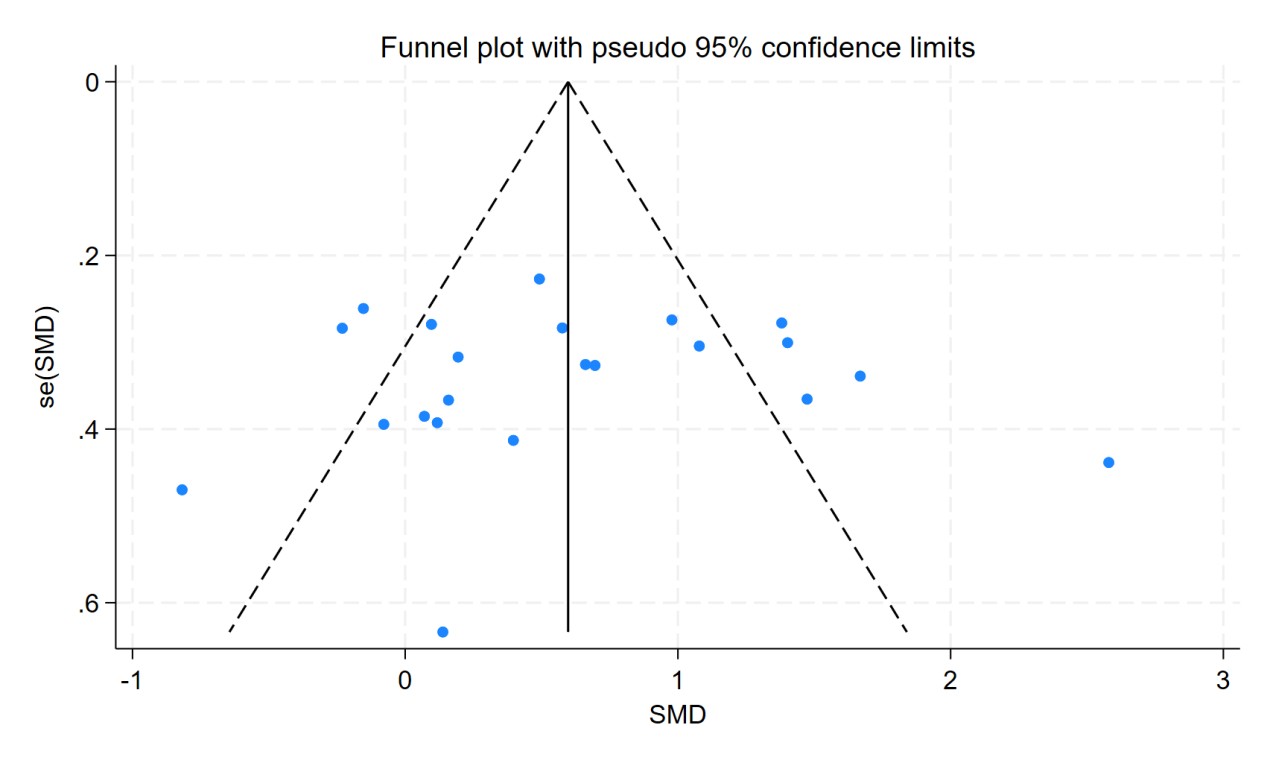


Figure S21 Funnel plot of the meta-analysis of FMA-UE


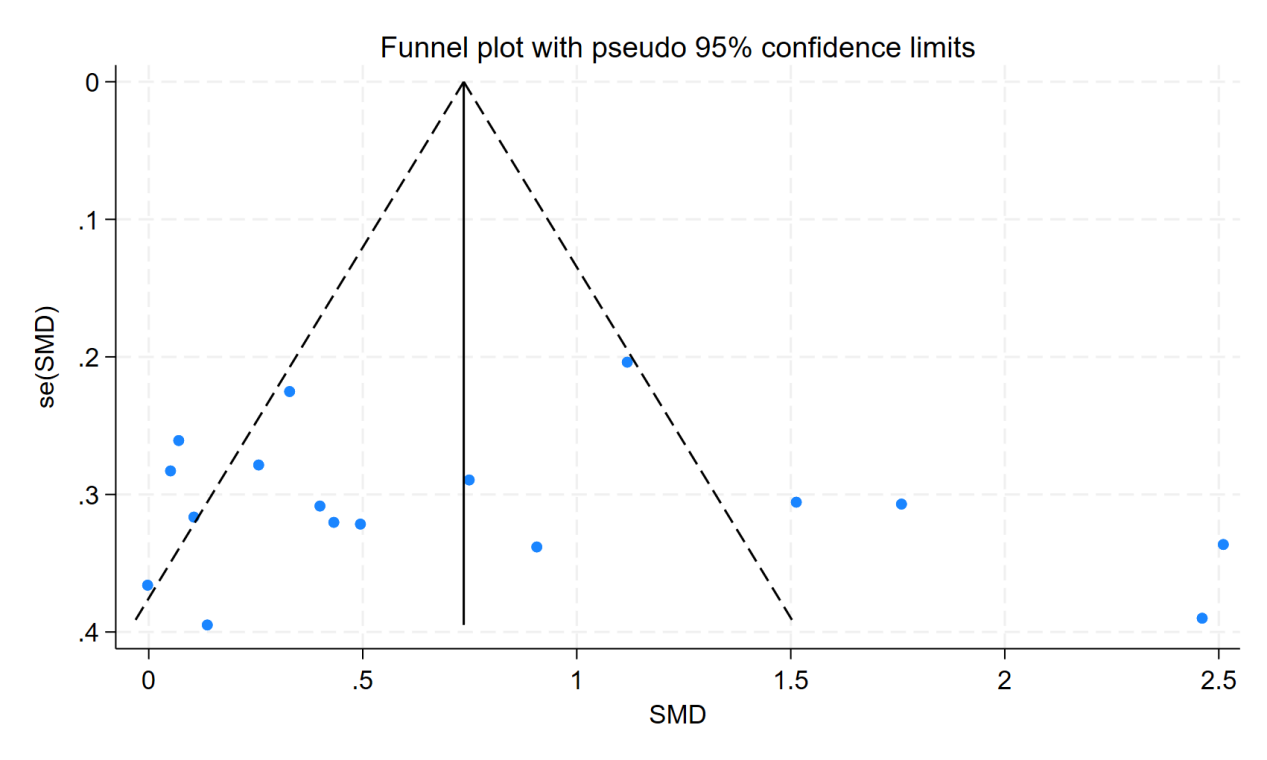


Figure S22 Funnel plot of the meta-analysis of Barthel Index


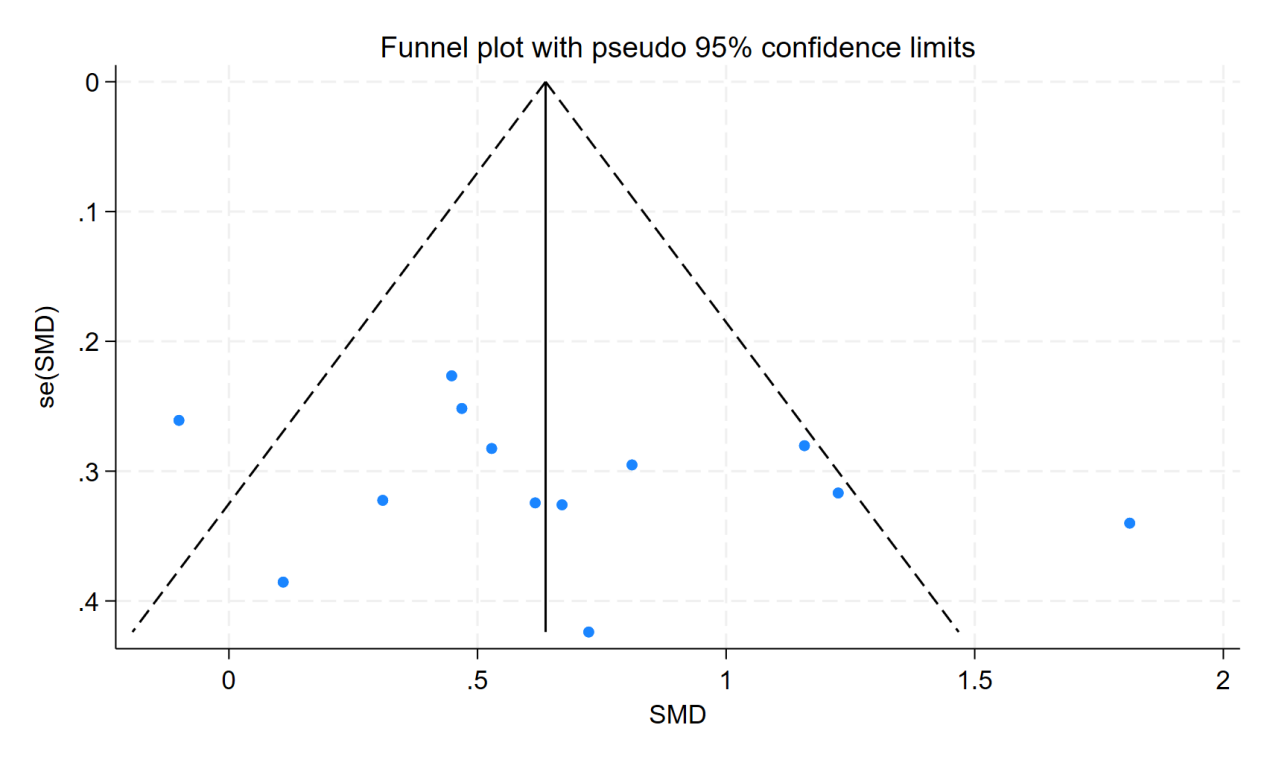


Figure S23 Funnel plot of the meta-analysis of ARAT


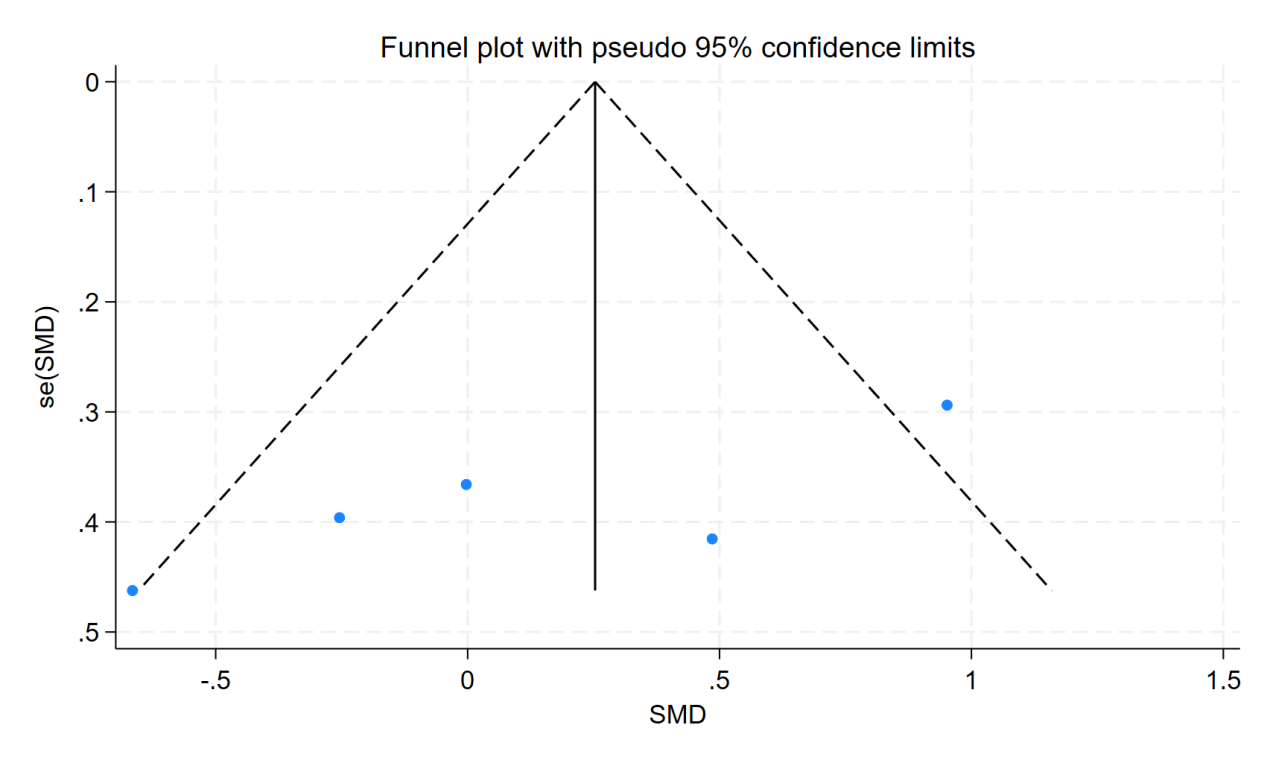


Figure S24 Funnel plot of the meta-analysis of BBT


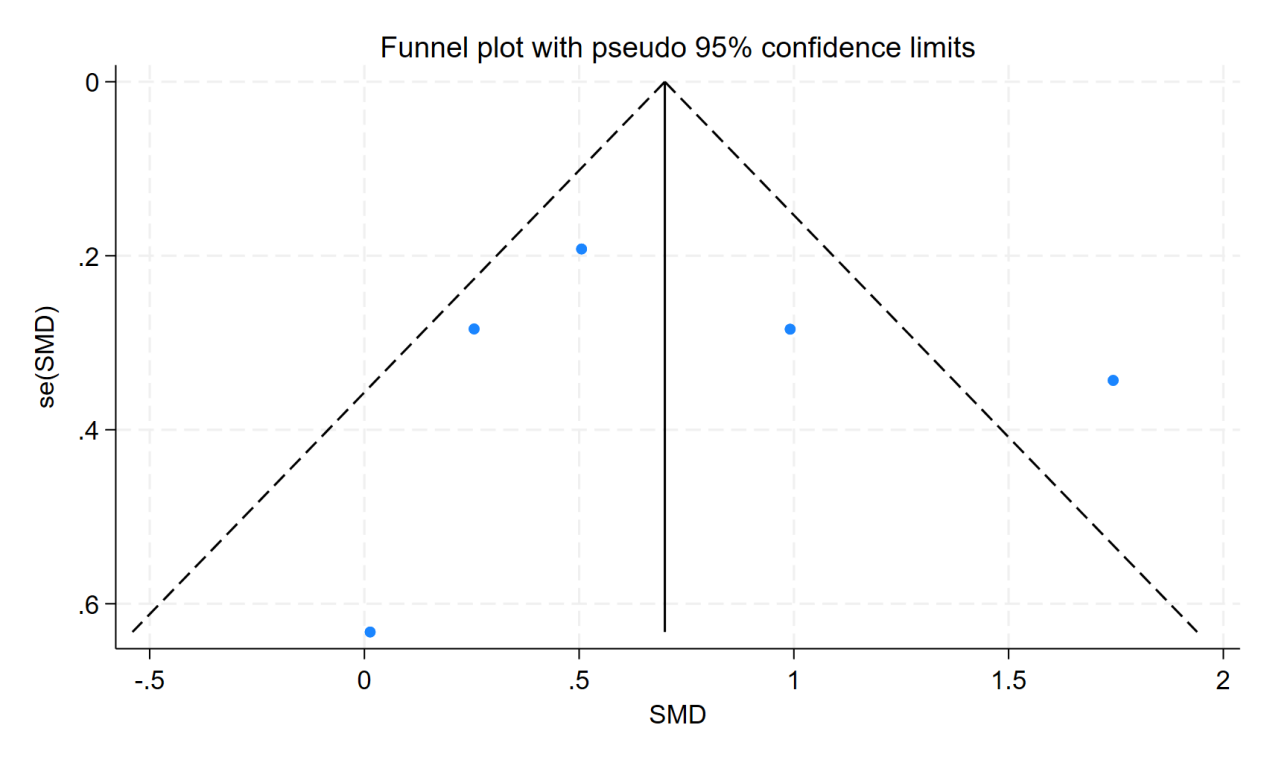


Figure S25 Funnel plot of the meta-analysis of WMFT


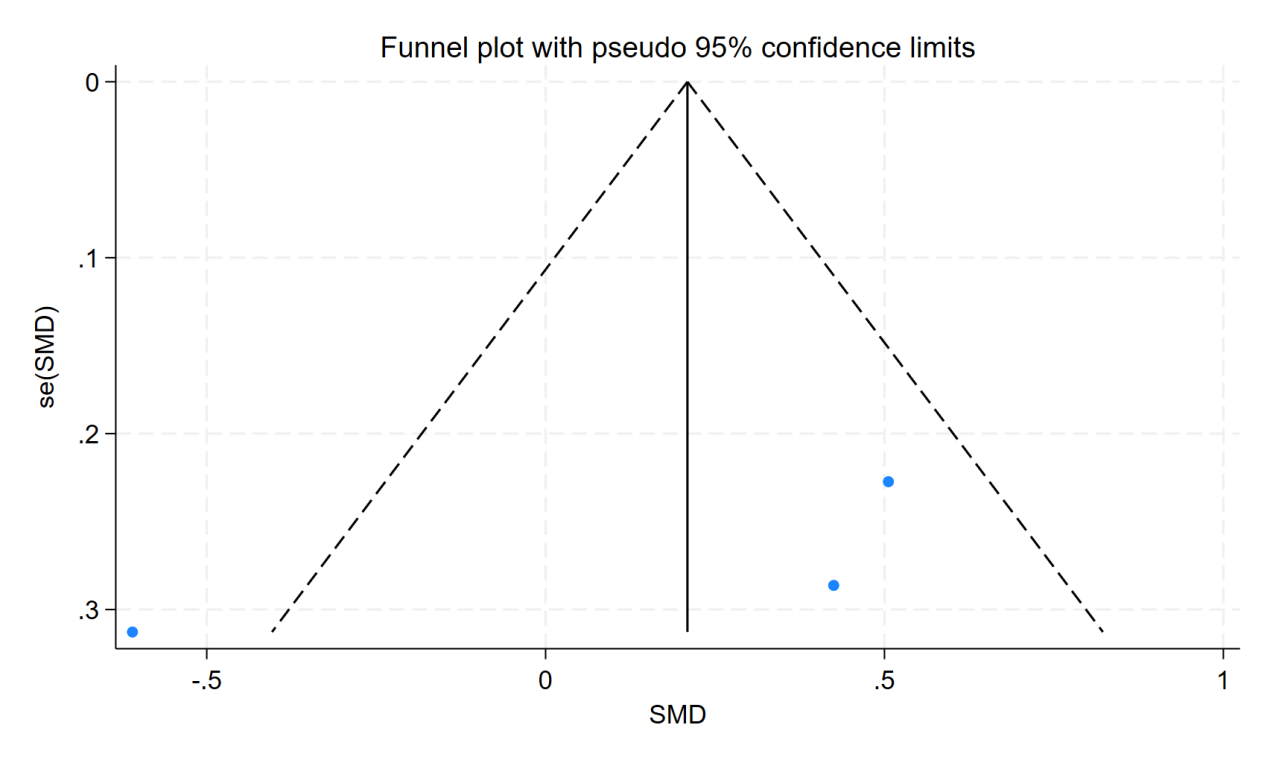


Figure S26 Funnel plot of the meta-analysis of MAS


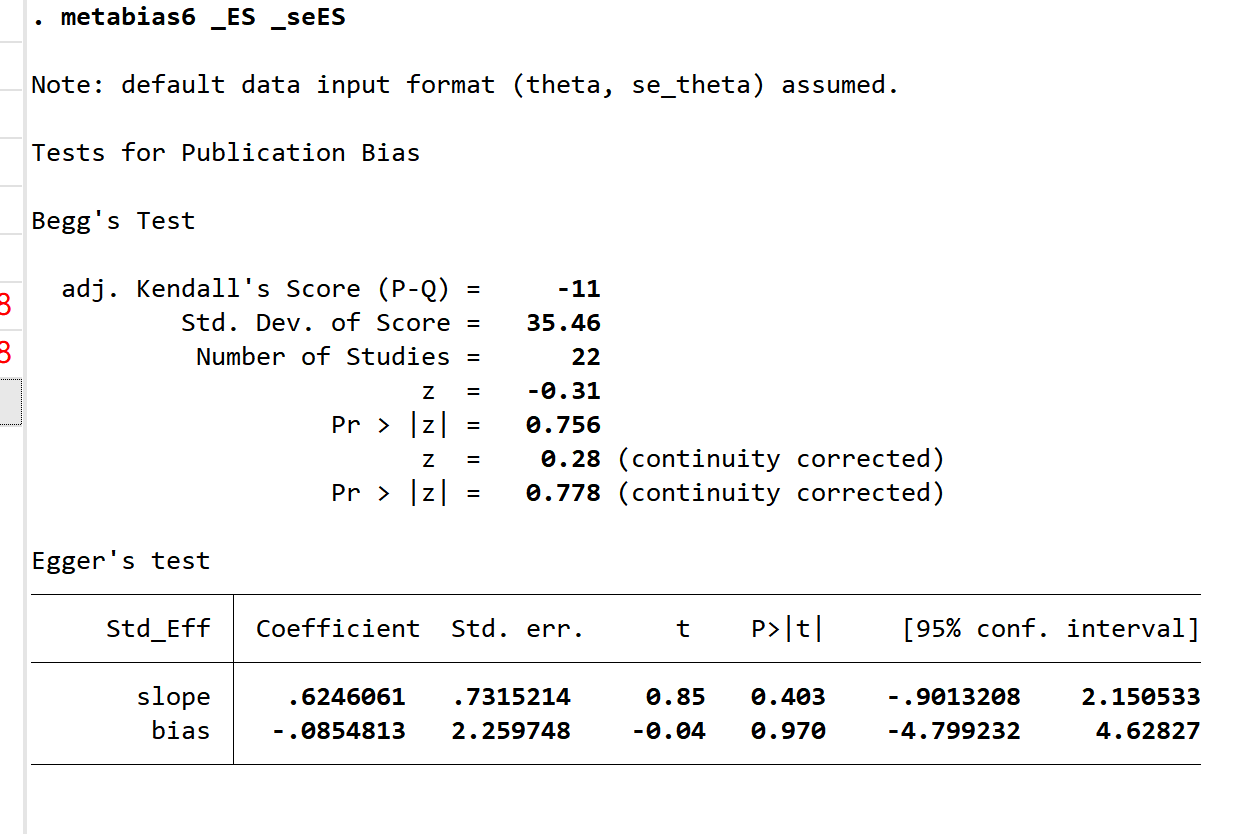


Figure S27 Egger’s test for FMA-UE


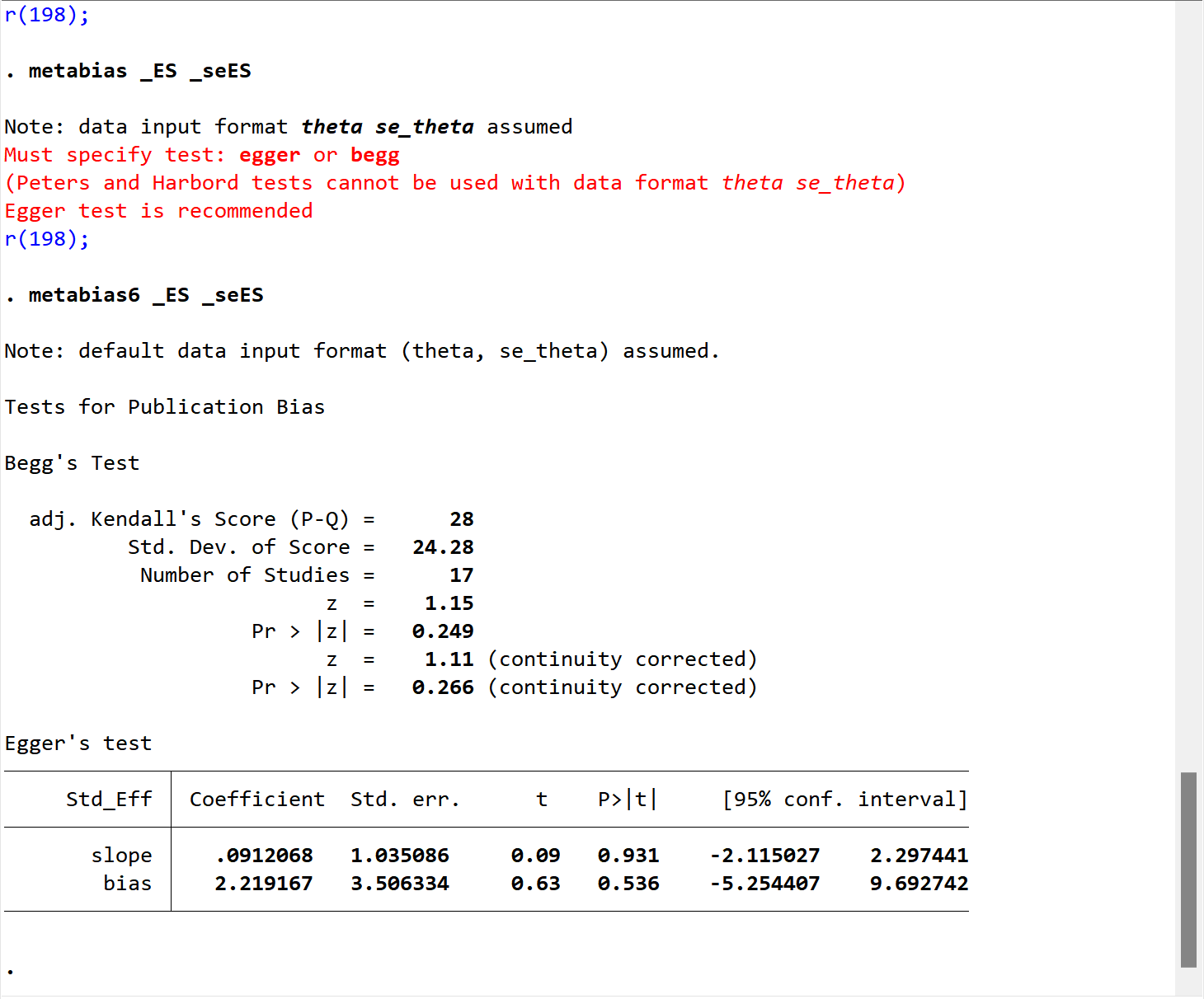


Figure S28 Egger’s test for Barthel Index


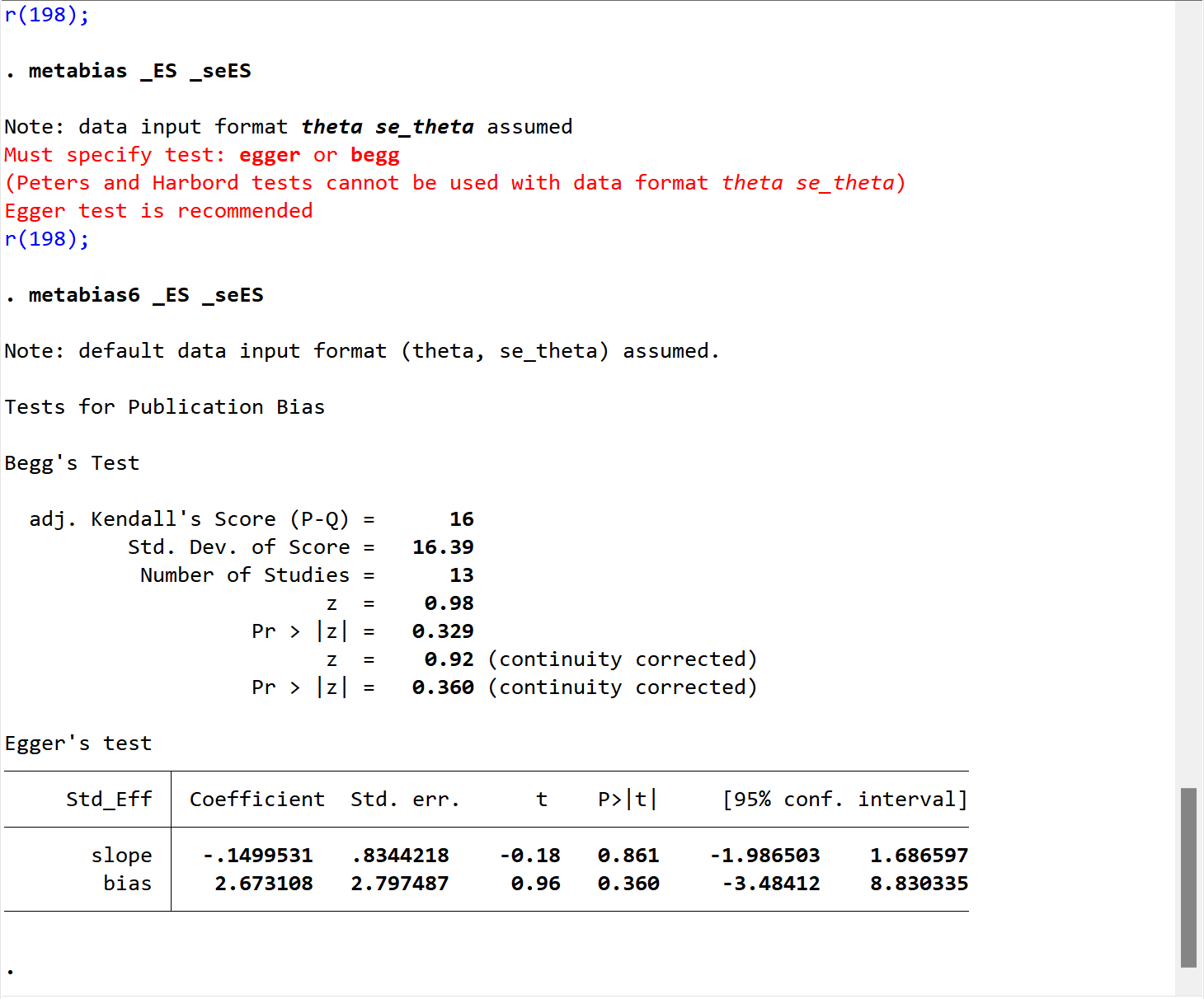


Figure S29 Egger’s test for ARAT


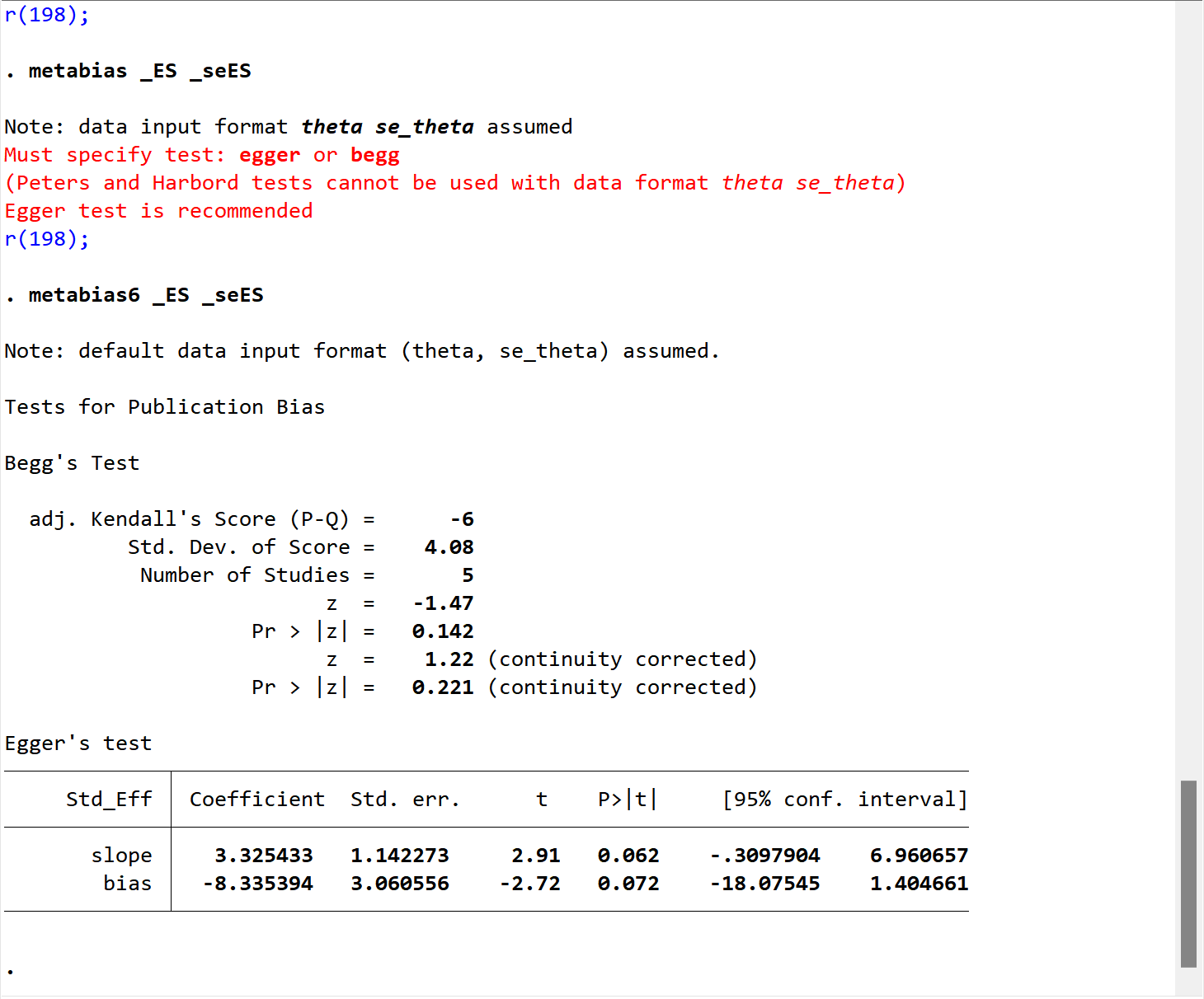


Figure S30 Egger’s test for BBT


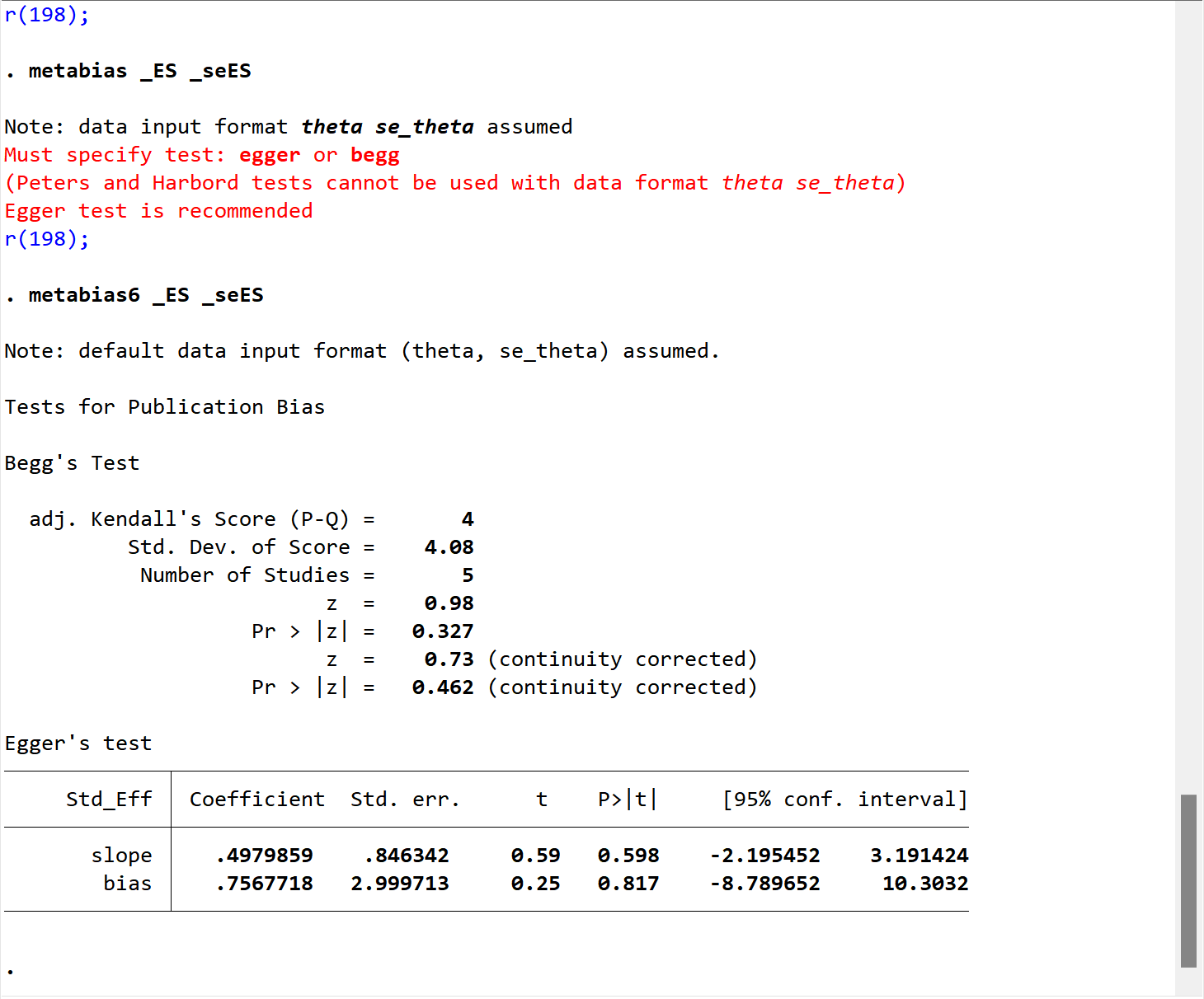


Figure S31 Egger’s test for WMFT


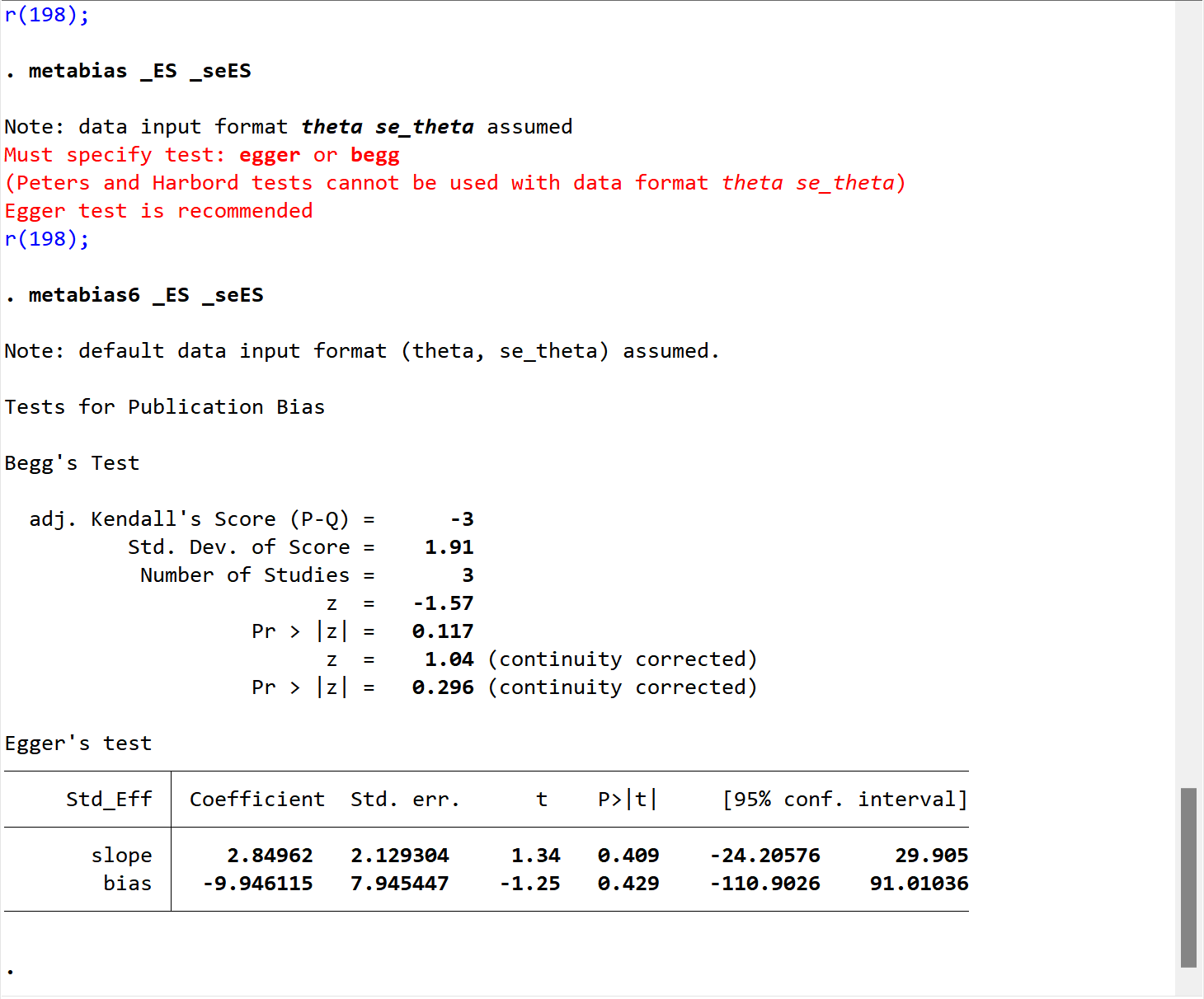


Figure S32 Egger’s test for MAS
